# Single-cell O_2_ exchange imaging shows that cytoplasmic diffusion is a dominant barrier to efficient gas transport in red blood cells

**DOI:** 10.1073/pnas.1916641117

**Published:** 2020-04-22

**Authors:** Sarah L. Richardson, Alzbeta Hulikova, Melanie Proven, Ria Hipkiss, Magbor Akanni, Noémi B. A. Roy, Pawel Swietach

**Affiliations:** ^a^Department of Physiology, Anatomy & Genetics, University of Oxford, OX1 3PT Oxford, England;; ^b^Molecular Haematology Laboratory, Oxford University Hospitals National Health Service (NHS) Foundation Trust, John Radcliffe Hospital, OX3 9DS Oxford, England;; ^c^Haematology, Milton Keynes University Hospital, MK6 5LD Milton Keynes, England;; ^d^Weatherall Institute of Molecular Medicine & National Institute for Health Research (NIHR) Oxford Biomedical Research Center (BRC) Haematology Theme, University of Oxford, OX3 9DS Oxford, England

**Keywords:** erythrocyte, diffusion, oxygen, fluorescence, gas channels

## Abstract

Disorders of oxygen transport are commonly attributed to inadequate carrying capacity (anemia) but may also relate to inefficient gas exchange by red blood cells (RBCs), a process that is poorly characterized yet assumed to be rapid. Without direct measurements of gas exchange at the single-cell level, the barriers to O_2_ transport and their relationship with hematological disorders remain ill defined. We developed a method to track the flow of O_2_ in individual RBCs by combining ultrarapid solution switching (to manipulate gas tension) with single-cell O_2_ saturation fluorescence microscopy. O_2_ unloading from RBCs was considerably slower than previously estimated in acellular hemoglobin solutions, indicating the presence of diffusional barriers in intact cells. Rate-limiting diffusion across cytoplasm was demonstrated by osmotically induced changes to hemoglobin concentration (i.e., diffusive tortuosity) and cell size (i.e., diffusion pathlength) and by comparing wild-type cells with hemoglobin H (HbH) thalassemia (shorter pathlength and reduced tortuosity) and hereditary spherocytosis (HS; expanded pathlength). Analysis of the distribution of O_2_ unloading rates in HS RBCs identified a subpopulation of spherocytes with greatly impaired gas exchange. Tortuosity imposed by hemoglobin was verified by demonstrating restricted diffusivity of CO_2_, an acidic gas, from the dissipative spread of photolytically uncaged H^+^ ions across cytoplasm. Our findings indicate that cytoplasmic diffusion, determined by pathlength and tortuosity, is a major barrier to efficient gas handling by RBCs. Consequently, changes in RBC shape and hemoglobin concentration, which are common manifestations of hematological disorders, can have hitherto unrecognized and clinically significant implications on gas exchange.

## Significance

Blood is routinely tested for gas-carrying capacity (total hemoglobin), but this cannot determine the speed at which red blood cells (RBCs) exchange gases. Such information is critical for evaluating the physiological fitness of RBCs, which have very limited capillary transit times (<1 s) for turning over substantial volumes of gas. We developed a method to quantify gas exchange in individual RBCs and used it to show that restricted diffusion, imposed by hemoglobin crowding, is a major barrier to gas flows. Consequently, hematological disorders manifesting a change in cell shape or hemoglobin concentration have uncharted implications on gas exchange, which we illustrate using inherited anemias. With its single-cell resolution, the method can identify physiologically inferior subpopulations, providing a clinically useful appraisal of blood quality.

Red blood cells (RBCs) are highly adapted morphologically, biochemically, and physiologically to exchange large volumes of O_2_ and CO_2_ during a capillary sojourn of less than 1 s (1). Impairment to any one of these adaptations can compromise gas exchange at the lungs and systemic tissues to the detriment of whole-body physiology. Blood samples taken as part of clinical care are routinely tested for indices of O_2_-carrying capacity, such as total hemoglobin (Hb) concentration, hematocrit, or mean corpuscular hemoglobin concentration (MCHC), but these do not supply information pertaining to the kinetics of gas turnover. Indeed, impaired gas (un-)loading from RBCs can be as damaging as anemia but come undetected using routinely used blood tests.

Historically, our understanding of O_2_ handling by RBCs has been based on reaction rates measured in acellular Hb solutions. However, the intact RBC imposes additional structural barriers, such as membrane permeation and cytoplasmic diffusion (2). Due to their naturally rapid pace, gas fluxes in and out of RBCs are technically challenging to capture, and consequently, the rate-limiting steps for gas exchange are difficult to identify. In particular, measurements of gas diffusion inside intact RBCs are lacking, although it is widely assumed that this is a rapid process (3–7). In contrast, permeation across the membrane bilayer has been postulated to be rate limiting for gas flows (2), and a parallel pathway in the form of gas channels (8) is considered necessary for efficient gas exchange (9–13). If, contrary to this paradigm, resistance to gas flow was imposed mainly by the cytoplasm, then gas exchange would be exquisitely sensitive to cell shape (diffusion pathlength) and MCHC (tortuosity imposed by Hb “crowding”) (14, 15), with major implications for our understanding of RBC disorders. A comprehensive appraisal of gas handling must also consider RBC heterogeneity because many hematological disorders manifest a spectrum of phenotypes among circulating cells. Pooled population measurements of gas exchange inherently lack single-cell resolution and hence fail to detect subpopulations, which may be relevant to the management of blood diseases. Indeed, the clinical value of measuring the statistical distribution of an RBC parameter has been demonstrated for corpuscular volume, which has a wider distribution width in iron deficiency and certain thalassemias (16, 17).

For the purpose of this study, we developed a method for tracking gas exchange in RBCs at single-cell resolution. We apply this method to measure O_2_ (un-)loading in individual RBCs, describe the statistical distribution of gas exchange kinetics in a population of RBCs, and characterize the barriers that restrict the flow of respiratory gases. We tested evidence for rate-limiting diffusion by subjecting wild-type RBCs to osmotically induced changes in cell volume and Hb concentration and by studying RBC disorders in which diffusive properties are altered. Hemoglobin H (HbH) disease is an alpha-thalassemia commonly arising from the deletion of three alpha genes. It is characterized by microcytic and hypochromic RBCs (18), circumstances that would favor faster cytoplasmic diffusion rates, but also increased membrane rigidity (19), which should restrict membrane permeability (20). Hereditary spherocytosis (HS) is the most common inherited anemia with a prevalence of 1:2,000 in northern Europeans (21, 22). Here, defective surface anchoring of the cytoskeleton causes cells to progressively attain a spherical shape with expanded cytoplasmic pathlength for diffusing gases (21, 23). We illustrate the utility of making single-cell measurements by describing the kinetic heterogeneity of RBCs in HS blood. As the spleen extracts aberrantly shaped cells, blood from HS patients will contain a mixture of spherical and disk-shaped cells (24, 25). Although the physical characteristics of these subpopulations are known, their gas exchange kinetics are implicitly assumed to be adequate. This supposition underpins the rationale for interventions, such as splenectomy, which reverses anemia by withholding spherocytes. If, however, these “rescued” cells were found to be physiologically inadequate for exchanging O_2_, their systemic retention may be less beneficial than anticipated.

Herein, we present a single-cell method for tracking gas handling by RBCs and demonstrate its scientific and clinical value by describing the rate-limiting steps to efficient gas exchange and relating these to hematological disorders.

## Results

### O_2_ Exchange Can Be Followed in an Individual RBC by Single-Cell O_2_ Saturation Imaging.

The appropriate technique for measuring O_2_ handling by RBCs must satisfy two criteria: 1) produce rapid and well-controlled changes in O_2_ tension immediately outside the RBCs so that the maneuver itself does not artifactually set the rate of the biological response and 2) dynamically track the O_2_ saturation response in an individual RBC.

**Fig. 1.**
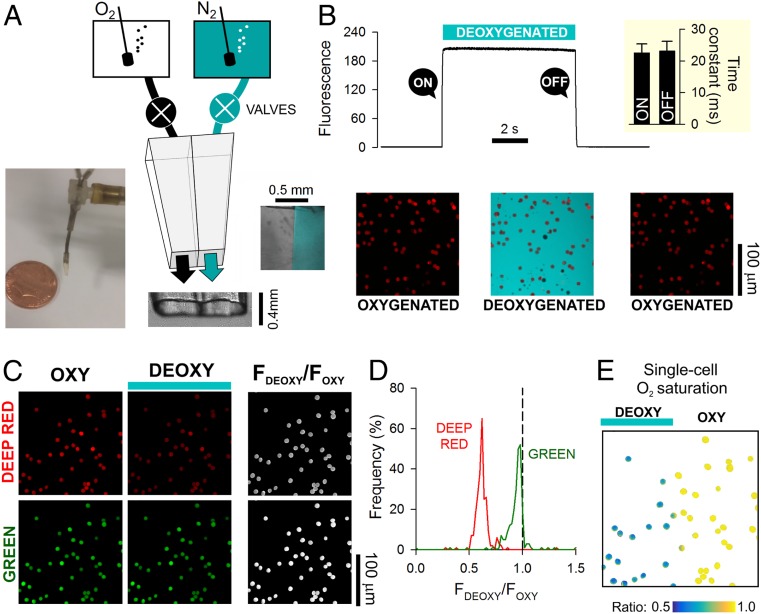
Measuring O_2_ exchange in RBCs using single-cell O_2_-saturating imaging. (*A*) Schematic of a dual microperfusion device loaded with oxygenated and deoxygenated solutions. *Inset* shows both microstreams released in parallel, one of which was labeled with 30 µM fluorescein. When flows are calibrated and balanced, microstreams become clearly separated. (*B*) Ultrarapid switching between the microstreams. Hypoxic microstream was labeled with 30 µM fluorescein. Rate of solution switching can be estimated from the fluorescence signal surrounding RBCs (wild type) loaded with CellTracker Deep-Red. Switching between solutions can be achieved with a time constant of 23 ms (*Inset*; *n* = 5). (*C*) Wild-type RBCs loaded with a mixture of Deep-Red and Green, showing fluorescence in oxygenated and deoxygenated microstreams. For these experiments, neither solution was fluorescently labeled. Grayscale ratio maps (*Right*) were calculated from these fluorescence maps (*Left* and *Center*). (*D*) On deoxygenation, Deep-Red fluorescence is reduced, whereas Green remains constant (wild-type RBCs; *n* = 356 cells). (*E*) Illustration of the method’s ability to resolve differences in O_2_ saturation. The field of view was split into oxygenated and deoxygenated halves by releasing both microstreams in parallel; only cells in the deoxygenated compartment showed a decrease in Deep-Red/Green ratio.

To address the first requirement, a double-barreled, square-bore (0.4-mm) microperfusion device (Fig. 1*A*) was used to rapidly alternate between an oxygenated microstream (bubbled with 100% O_2_) and a deoxygenated (anoxic) microstream (bubbled with 100% N_2_ and containing the O_2_ scavenger dithionite; 1 mM). Experiments were performed in the absence of CO_2_ to simplify data interpretation. To determine the speed of solution switching, the deoxygenated solution was labeled with 30 µM fluorescein (excitation 488 nm, emission 500 to 550 nm), and the device was placed upstream to RBCs that had been preloaded with CellTracker Deep-Red and settled at the base of a glass coverslip. Switching to the fluorescein-labeled microstream produced a sharp increase in fluorescence immediately outside cells following a very fast time constant of 23 ms (Fig. 1*B*).

The second criterion was met by exploiting O_2_-dependent changes in the optical properties of Hb. Upon oxygenation, absorbance of Hb in the 650- to 700-nm range decreases relative to that in the 500- to 550-nm range. Thus, O_2_ saturation of an individual RBC could be interrogated using a combination of two fluorescent dyes that emit in these ranges. A suitable pair of dyes is CellTracker Deep-Red and CellTracker Green (Fig. 1*C*), which produce no significant bleed through when excited by 633- and 488-nm lasers simultaneously. RBCs, loaded with CellTracker Deep-Red and CellTracker Green, were plated on a glass coverslip at the base of an inspection chamber and superfused with 4-(2-hydroxyethyl)-1-piperazineethanesulfonic acid (Hepes)-buffered solution (3 mL/min). The dual microperfusion device, placed upstream of cells, delivered a Hepes-buffered microstream at ∼8 µL/s, producing a linear velocity of 50 mm/s at the outlet of the pipette. Such laminar flow produces a modest shear rate of ∼10^2^ s^−1^ (velocity/height of the stream), which is below the level that normally leads to complex deformations (e.g., parachute or polylobe) of RBCs in the microcirculation (26, 27). O_2_ unloading was triggered by switching from the oxygenated to deoxygenated microstream. This maneuver reduced Deep-Red fluorescence to a greater degree than Green fluorescence (Fig. 1*D*), and therefore, the fluorescence ratio provides a dynamic readout of Hb O_2_ saturation. To illustrate the versatility of this method, the dual microperfusion device was configured to release both microstreams in parallel, which sharply divided RBCs in the field of view according to oxygenation status (Fig. 1*E*). In this protocol, the response of Deep-Red to anoxia relates to Hb O_2_ unloading rather than an unreported effect of O_2_ on the dye, as no change in fluorescence ratio was observed in cell lines that do not produce Hb (*SI Appendix*, Fig. S1).

### O_2_ Unloading from Wild-Type RBCs Is Slower than Previously Estimated.

**Fig. 2.**
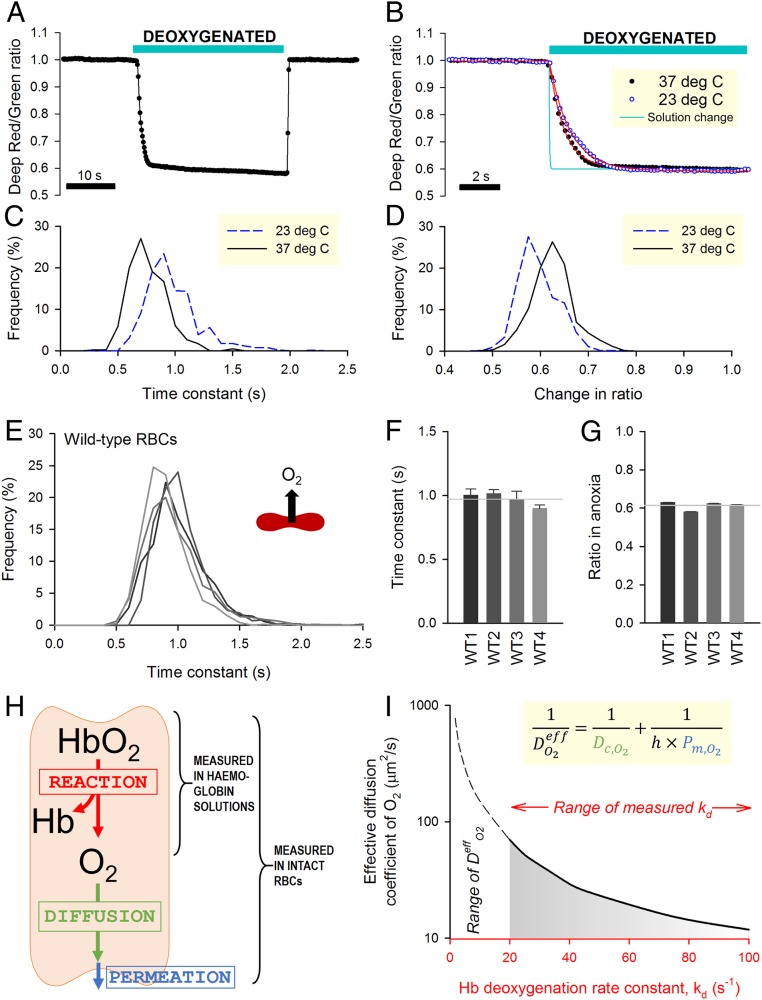
Measuring O_2_ exchange with single-cell resolution in wild-type (WT) RBCs. (*A*) Time course of O_2_ saturation during a 30-s exposure to deoxygenated solution (averaged for 10 cells within a single field of view). (*B*) Detail of unloading kinetics superimposed on the time course of solution exchange (Fig. 1*B*). Red lines are best-fit monoexponential curves. (*C*) Frequency histogram of the time constant of O_2_ unloading (τ_O2_) and (*D*) overall change of fluorescence ratio measured at either 23 °C or 37 °C. (*E*) Histogram of τ_O2_ in WT RBCs from four donors (WT1 to WT4) determined at 23 °C. (*F*) Mean ± variance for τ_O2_ and (*G*) overall fluorescence ratio response (*n* = 702, 738, 1,489, 751). Gray lines show means for WT1 to WT4. (*H*) Schematic of the diffusion–reaction system that describes O_2_ unloading from RBCs. (*I*) Graphical solution to the diffusion–reaction equation shows the combinations of effective O_2_ diffusivity (D^eff^_O2_) and Hb–O_2_ dissociation rate constant (k_d_) that fit τ_O2_ data for WT RBCs (0.971 s). *Inset* shows the equation that defines D^eff^_O2_ in terms of membrane permeation (P_m,O2_), mean diffusion distance (*h*), and cytoplasmic diffusivity (D_c,O2_). Simulation for WT RBC: h = 0.885 µm (half-thickness of RBC). Considering the experimentally derived range of k_d_ (20 to 100 s^−1^), D^eff^_O2_ is <70 µm^2^/s.

Fig. 2*A* shows an exemplar time course of O_2_ saturation measured in RBCs during controlled changes in extracellular O_2_ tension. The kinetics of O_2_ unloading can be expressed in terms of a time constant (τ_O2_) calculated by best fitting to a monoexponential curve (Fig. 2*B*). Since these measurements are performed on a cell-by-cell basis, the statistical distribution of τ_O2_ can be obtained for the imaged cohort (Fig. 2*C*). Time courses can also be analyzed in terms of the overall decrease in ratio, which relates to the amount of oxygen released (Fig. 2*D*). The rate of O_2_ unloading was ∼30% faster at 37 °C compared with room temperature (23 °C), a difference that is consistent with the thermodynamics diffusion phenomena (28). To determine the rate of O_2_ unloading from wild-type RBCs, measurements were undertaken on blood samples from four control individuals (Fig. 2*E*). To improve temporal resolution, these experiments were performed at 23 °C. Data were acquired from at least five fields of view and five independent dye loadings in order to collect adequate data for constructing frequency histograms of τ_O2_. Mean τ_O2_ was similar in all control samples (Fig. 2*F*), and the population variance of τ_O2_ was relatively small. The overall decrease in fluorescence ratio upon deoxygenation was, on average, 39% and showed narrow variation (Fig. 2*G*).

In wild-type RBCs, mean τ_O2_ was 0.971 s, which is strikingly slow, particularly in relation to a typical capillary transit time. Indeed, previous measurements on acellular Hb solutions had determined O_2_ unloading to follow a substantially faster time constant of 20 to 100 ms at matching temperature (23 °C) (3, 29). This discrepancy can be explained by the presence of additional barriers that exist uniquely in the cellular context, notably diffusion through cytoplasm and permeation across the surface membrane. The resistance imposed collectively by these barriers can be quantified using a mathematical model describing O_2_ unloading in terms of reaction, diffusion, and permeation (Fig. 2*H*). Using kinetic data for Hb–O_2_ binding, it is thus possible to estimate the effective diffusion coefficient (D^eff^_O2_), which lumps membrane permeation (P_m,O2_), cytoplasmic diffusion (D_c,O2_), and mean diffusion distance (h) as follows:

$${\left({\text{D}}_{{\text{O}}_{2}}^{\text{eff}}\right)}^{-1}={\left({\text{D}}_{\text{c},{\text{O}}_{2}}\right)}^{-1}+{\left(\text{h}\times {\text{P}}_{\text{m},{\text{O}}_{2}}\right)}^{-1}.$$ [1]

Unbinding of the first O_2_ molecule from oxyhemoglobin follows a rate constant (k_d_) of 20 to 30 s^−1^ (3, 29), but subsequent unbinding steps are faster due to cooperativity, resulting in an ensemble k_d_ as fast as ∼100 s^−1^. A mathematical representation of the Hb–O_2_ reaction (30) (equations in *SI Appendix*) was implemented to derive the combinations of D^eff^_O2_ and k_d_ that fit τ_O2_ measurements. To solve this problem for τ_O2_ = 0.971 s and k_d_ > 20 s^−1^, D^eff^_O2_ was determined to be <70 µm^2^/s (Fig. 2*I*) (i.e., <5% of O_2_ diffusivity in water) (31).

### Cytoplasmic Diffusion Is a Substantial Barrier to Cellular Gas Exchange.

**Fig. 3.**
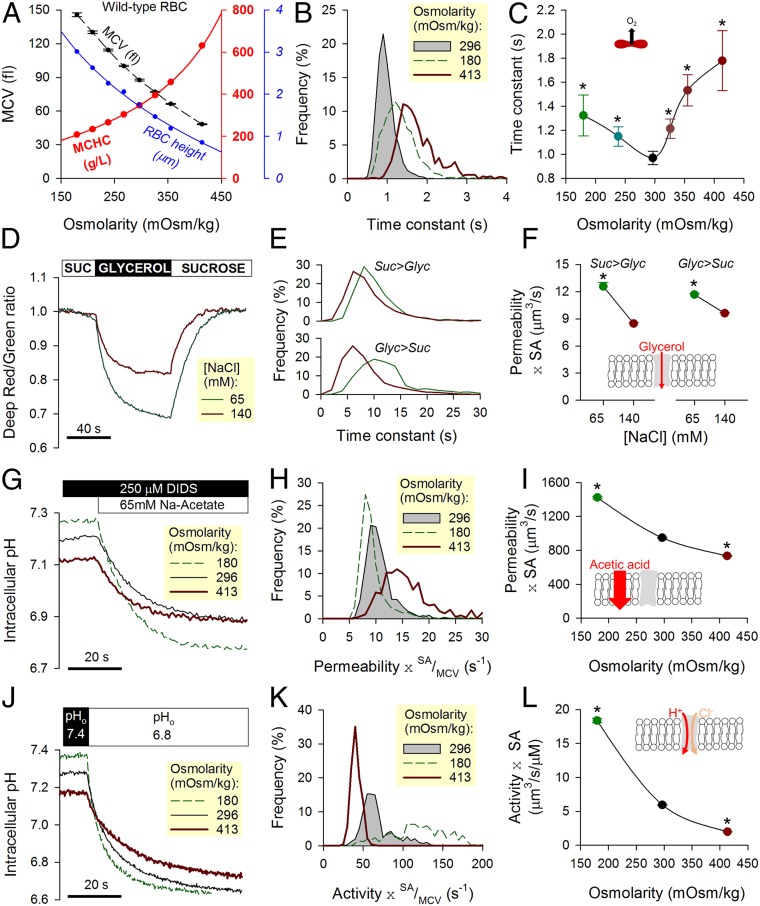
Effect of osmotic manipulations on transport properties in wild-type (WT) RBCs. (*A*) Changes in osmolarity brought about by adding or removing superfusate NaCl affect MCV, MCHC, and cell height (ratio of MCV to cell area). Mean ± SEM. (*B*) Both hypo- and hyperosmotic conditions slow the process of O_2_ unloading, which is quantified in terms of time constant τ_O2_. (*C*) Biphasic relationship between osmolarity and τ_O2_, with a nadir at physiological osmolarity. Mean ± variance (*n* = 3,688, 1,002, 3,680, 1,609, 895, and 841 from WT3 and WT4). (*D*) Deep-Red/Green ratio response to reversible cell swelling triggered by aquaporin-mediated glycerol influx under constant tonicity (100 mM glycerol replacing 100 mM sucrose). Experiments were performed under hypo- (+65 mM NaCl) and hypertonic (+140 mM NaCl) conditions. (*E*) Time constant of fluorescence ratio changes in response to glycerol influx (*Upper*) and efflux (*Lower*). (*F*) Glycerol permeability increases under hypotonic conditions. Mean ± SEM (*n* = 511 and 1,030 from WT3 and WT4). Equilibrium MCVs were 79 fL in 140 mM NaCl + 100 mM sucrose, 86 fL in 140 mM NaCl + 100 mM glycerol, 123 fL in 65 mM NaCl + 100 mM sucrose, and 148 fL in 65 mM NaCl + 100 mM glycerol. (*G*) pH_i_ imaged in cSNARF1-loaded RBCs during a protocol that measures permeability to acetic acid. Cells were rapidly exposed to Na-acetate (65 mM) in the presence of DIDS (250 µM) to block AE1 activity. The rate of pH_i_ change is a readout of acetic acid entry, which was measured over a range of osmolarity. (*H*) Hypo- and hyperosmotic conditions modestly affect membrane permeability to acetic acid. (*I*) A decrease in osmolarity modestly increases the product of acetic acid permeability and SA. Mean ± SEM (*n* = 930, 1,658, 1,500 from WT3 and WT4). (*J*) pH_i_ imaged in cSNARF1-loaded RBCs during a protocol that measures AE1 activity. Extracellular pH was rapidly dropped from 7.4 to 6.8, and the rate of pH_i_ change provides a readout of Cl^−^/OH^−^ exchange, which was probed over a range of osmolarity. (*K*) Hypo- and hyperosmotic conditions affect AE1 activity. (*L*) A decrease in osmolarity increases the product of AE1 activity coefficient and SA. Mean ± SEM (*n* = 426, 730, 662 from WT3 and WT4). *Significant difference to physiological osmolarity.

Low D^eff^_O2_ could arise from a combination of slow membrane permeation (P_m,O2_) and slow cytoplasmic diffusion (D_c,O2_). For example, unhindered cytoplasmic diffusivity would indicate a membrane permeability less than ∼80 µm/s (Eq. **1**), but if cytoplasmic diffusivity was restricted, P_m,O2_ must be accordingly higher. To investigate the dominant barrier to gas flow, the diffusive properties of RBCs were manipulated experimentally by changing extracellular osmolarity (adding or removing NaCl) within a range that does not result in substantial hemolysis. An increase in osmolarity reduces mean corpuscular volume (MCV), concentrates Hb (MCHC), and constricts cell thickness calculated as the quotient of MCV to cell area (Fig. 3*A*). Cell area was calculated from the outline of CellTracker fluorescence in RBCs and calibrated using fluorescent beads. These beads were also used to calibrate flow cytometric measurements of MCV. If gas transport were meaningfully obstructed by cytoplasm, then τ_O2_ would be highly sensitive to changes in MCHC and cell thickness (i.e., diffusive tortuosity and diffusion pathlength, respectively). Indeed, O_2_ unloading measured over a range of osmolarity (Fig. 3*B*) followed a biphasic relationship with a nadir (i.e., fastest exchange) near physiological osmolarity (Fig. 3*C*). Slower gas flows under hypoosmotic conditions are consistent with expanded pathlengths in swollen cells; in contrast, hyperosmotic conditions may obstruct O_2_ exchange by the increase in Hb density. The relationship in Fig. 3*C* predicts that a condition with modestly reduced MCHC and dilated pathlength should produce slower O_2_ exchange. This was confirmed in RBCs from donors with mild to moderate iron deficiency (*SI Appendix*, Fig. S2), where a change in MCHC and cell thickness (equivalent to a mild hypoosmotic swelling of wild-type cells) slowed O_2_ unloading by 12%. Notwithstanding these inferences about the role of cytoplasmic diffusion in setting the rate of O_2_ unloading, an effect of osmolarity on membrane permeability properties cannot be excluded. There are two major routes for O_2_ entry across membranes—the lipid bilayer and putative gas channels—and their responses to osmotic stretch were interrogated by studying solute fluxes known to be rate limited by membrane permeation.

Since aquaporins are putative gas channels (8, 13), the first protocol measured glycerol permeability as a readout of aquaporin AQP3-mediated transport (32). Experimental maneuvers involved rapid switching between 100 mM sucrose and 100 mM glycerol, thereby maintaining a constant extracellular osmolarity (33) (Fig. 3*D*). These solutions also contained either 65 or 140 mM NaCl to vary overall osmolarity. Changes in cell volume evoked by glycerol fluxes were monitored from the Deep-Red/Green fluorescence ratio, which, at constant O_2_ tension, decreases upon cell swelling (Fig. 3*E*). The product of glycerol permeability and cell surface area (SA) was higher in hypotonically swollen RBCs (Fig. 3*F*), indicating that membrane stretch is likely to increase rather than restrict aquaporin conductance. The second protocol followed the entry of acetic acid across the lipid bilayer from the time course of intracellular pH (pH_i_). To block the activity of Anion Exchanger 1 (AE1), which could influence pH_i_, DIDS (4,4′-diisothiocyano-2,2′-stilbenedisulfonic acid; 250 µM) was added to solutions. Acetic acid was delivered to RBCs by rapid solution switching, and changes in pH_i_ were imaged using the fluorescent dye cSNARF1 loaded into cells (Fig. 3*G*). The ratio of the overall pH_i_ change (related to buffering capacity) (*SI Appendix*, Fig. S3) and its time constant is equal to permeability multiplied by the SA/MCV ratio (Fig. 3*H*). Scaling by volume provides an appraisal of the lipid bilayer’s conductance to acetic acid (equations in *SI Appendix*). The membrane was found to be more permeable to acetic acid in osmotically swollen cells (Fig. 3*I*). The third protocol measured the activity of AE1, the most abundant ion transporter in the RBC membrane and, potentially, another pathway for gas entry. AE1-mediated transport was interrogated in cSNARF1-loaded RBCs by measuring the pH_i_ response to a rapid extracellular acidification, which triggers Cl^−^/OH^−^ exchange (Fig. 3*J*). The membrane’s AE1 transport capacity (Fig. 3*K* and equations in *SI Appendix*) increased steeply with osmotic swelling (Fig. 3*L*). We thus show that bilayer permeability (to acetic acid), aquaporin conductance (to glycerol), and AE1 activity are increased with hypotonic swelling. This general activatory effect of stretch on membrane permeability strongly suggests that the slowing of O_2_ exchange in hypotonically swollen RBCs (Fig. 3*C*) was not related to a tightening of membrane permeability to gases. Instead, the likely explanation for slower O_2_ transport is slow diffusion across an expanded cytoplasmic pathlength.

### Slow Cytoplasmic Gas Diffusivity Arises from the High Density of Hb.

To determine the extent to which cytoplasm restricts the flow of gas, a more precise quantification of intracellular diffusivity is necessary. This was obtained by measuring a diffusive process evoked within the cytoplasmic compartment. Conveniently, intracellular diffusion of CO_2_, an acidic gas, can be traced from spatial pH_i_ dynamics using a method that exploits the chemical equilibrium between CO_2_, HCO_3_^−^, and H^+^ (14). The principle underpinning this technique is that H^+^ ions are unable to diffuse freely in the highly buffered environment of RBC cytoplasm; instead, transport occurs solely aboard buffer molecules (i.e., facilitated diffusion). Consequently, a measured (apparent) H^+^ diffusion coefficient (D_H_^app^) is a readout of the diffusive properties of cytoplasmic buffers (14) (i.e., Hb and CO_2_/HCO_3_^−^) (*SI Appendix*, Fig. S3). Cytoplasmic CO_2_ diffusivity (D_c,CO2_) could thus be calculated by comparing D_H_^app^ measurements in the presence and absence of CO_2_/HCO_3_^−^ buffer (14, 28).

**Fig. 4.**
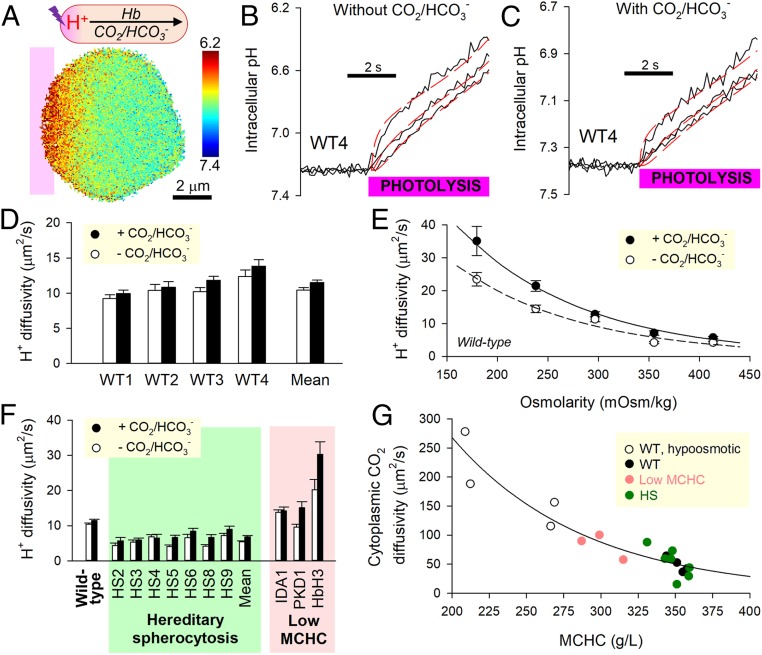
Measuring CO_2_ diffusivity in RBC cytoplasm. (*A*) Photolytic uncaging of H^+^ ions from NVA at one end of a wild-type (WT) RBC produces an acidic microdomain that dissipates diffusively across cytoplasm, facilitated by Hb and (if added) CO_2_/HCO_3_^−^ buffer. (*B*) Measurements in the absence of CO_2_/HCO_3_^−^; time course of pH_i_ (black trace) in 10 equally spaced ROIs across the width of a WT RBC. Traces for ROIs 1, 4, and 7 are shown for clarity. Best fit (red) to diffusion model for deriving H^+^ diffusivity, D_H_^app^. (*C*) Protocol performed under superfusion with CO_2_/HCO_3_^−^. (*D*) Summary data from four WT samples (*n* = 226 WT cells). (*E*) Relationship between osmolarity (varied by adding or removing NaCl) and D_H_^app^ in WT RBCs (*n* = 30 to 50 per bar). (*F*) Summary data from HS blood and three samples of reduced MCHC: iron deficiency (IDA1), puryvate kinase deficiency (PKD1), and HbH thalassemia (HbH3). Mean ± SEM (*n* = 20 to 35). (*G*) Relationship between MCHC and cytoplasmic CO_2_ diffusivity calculated from D_H_^app^ measured in the presence and absence of CO_2_/HCO_3_^−^.

Cytoplasmic H^+^ diffusion was triggered by photolytically uncaging H^+^ ions in one region of an RBC from the donor 6-nitroveratraldehyde (NVA; 1 mM) (Fig. 4*A*). To ensure that D_H_^app^ measurements relate to physiological pH_i_, starting pH_i_ was offset to an alkaline level by raising superfusate pH to 7.8. Under superfusion with CO_2_/HCO_3_^−^-free (Hepes-buffered) solutions, cytoplasmic H^+^ transport is facilitated by Hb molecules. The diffusive spread of H^+^ ions imaged in the horizontal plane from cSNARF1 fluorescence was slow in wild-type RBCs (Fig. 4*B*). Superfusion with CO_2_/HCO_3_^−^ introduces a parallel H^+^ shuttle into cytoplasm (Fig. 4*C*), and for these experiments, 12.5 µM DIDS was added to block membrane transport of HCO_3_^−^ by AE1 and thus, render the RBC a “closed” compartment with respect to H^+^ ions. If the diffusive capacity of intracellular CO_2_/HCO_3_^−^ was substantial, the addition of this buffer would have increased D_H_^app^ profoundly, but this was not observed (Fig. 4*D*). A previously described diffusion–reaction model for RBC H^+^ dynamics estimated D_c,CO2_ to be 48.9 ± 6.1 µm^2^/s (14), indicating substantially restricted diffusion relative to diffusivity in water (2,500 µm^2^/s). This degree of cytoplasmic obstruction is sufficient to explain slow O_2_ unloading without having to implicate another barrier, such as the membrane (Eq. **1**).

In order for short cytoplasmic distances to become significant barriers to gas exchange, the molecular components of RBCs must adequately obstruct the movement of gas. A likely candidate for this barrier function is Hb, which makes up ∼95% of the cell’s dry weight (34). This hypothesis was tested in RBCs that had been osmotically diluted or concentrated. D_H_^app^ measured in the presence or absence of CO_2_/HCO_3_^−^ increased at lower MCHC (Fig. 4*E*). Importantly, a more dilute macromolecular milieu allowed CO_2_/HCO_3_^−^ to become more effective at facilitating H^+^ transport. Consistent with this observation, RBCs from patients with reduced MCHC (iron deficiency anemia, pyruvate kinase deficiency, HbH thalassemia) supported faster D_H_^app^ (Fig. 4*F*).

It is plausible that the cytoskeleton may further influence gas movement, and this was explored in HS RBCs, which carry mutations in cytoskeletal elements. Hb-dependent H^+^ shuttling in HS RBCs was twofold lower than in wild-type cells but only modestly increased upon adding CO_2_/HCO_3_^−^. D_c,CO2_ in these cells was found to be 52.5 ± 9.4 µm^2^/s, i.e., not significantly different from wild-type RBCs and indicating that the molecular remodeling in HS does not alter CO_2_ diffusivity. In summary, the relationship between D_c,CO2_ and MCHC (plotted up to 360 g/L) shows that a “loosening” of Hb density allows greater diffusive freedom (Fig. 4*G*). Slower O_2_ unloading from osmotically swollen RBCs (Fig. 3*C*) must, therefore, be attributed to a lengthening of diffusive path to an extent that outweighs reduced crowding at dilute MCHC.

### Mercury Reduces Cytoplasmic Diffusion.

In some previous studies (8, 35), responses of gas exchange to mercurial substances were interpreted as evidence for channel-dependent gas permeation on the basis that conduits, such as aquaporins, are inhibited when reacted with Hg^2+^ (36). In RBCs, DIDS-sensitive Rh protein has also been proposed to conduct gases (11, 13). To test for these pathways, RBCs were pretreated with 100 µM DIDS and 250 µM Hg^2+^ for 10 min and assayed for O_2_ unloading rate (*SI Appendix*, Fig. S4*A*). The time constant for O_2_ unloading increased by a factor of three to 2.77 ± 0.39 s (*SI Appendix*, Fig. S4 *B* and *C*). However, this treatment also led to an increase in MCV (*SI Appendix*, Fig. S4*D*) and diffusion pathlength (*SI Appendix*, Fig. S4*E*). Additionally, Hg^2+^/DIDS reduced Hb-mediated H^+^ diffusion by a factor of two and obstructed H^+^ diffusivity in the presence of CO_2_/HCO_3_^−^ by a factor of three (*SI Appendix*, Fig. S4*F*). Taken together, the threefold slowing of O_2_ exchange in Hg^2+^/DIDS-treated RBCs can be explained by a more tortuous cytoplasmic environment for diffusion and an expanded pathlength without necessarily implicating changes to membrane properties.

### O_2_ Unloading Rate Is Altered in Disorders of RBC Shape and Hb Concentration.

**Fig. 5.**
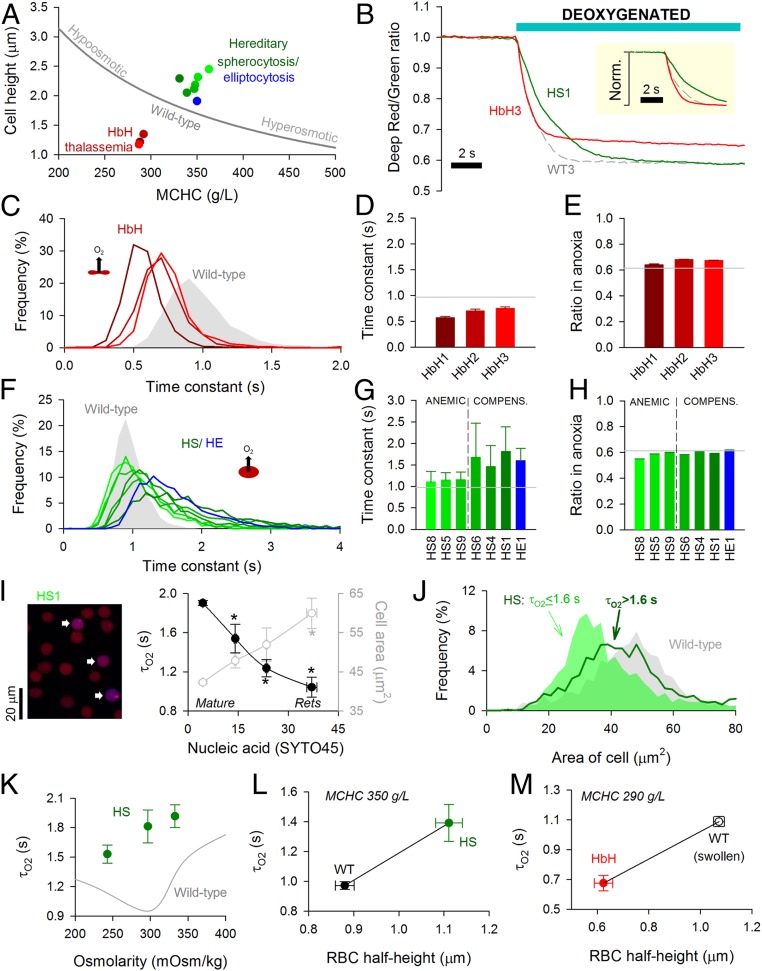
Disorders of RBC shape, size, and Hb affect O_2_ unloading. (*A*) Relationship between MCHC and cell height (thickness) for wild-type (WT) RBCs under various conditions of osmolarity (gray line) compared with data for HbH thalassemia (red), HS (green), and HE (blue). (*B*) Exemplar time course of O_2_ unloading in HbH, HS, and WT RBCs. (*Inset*) Traces normalized. (*C*) Frequency distribution of O_2_ unloading time constant (τ_O2_) for HbH blood. (*D*) Mean ± variance for τ_O2_ and (*E*) overall fluorescence ratio response in HbH RBCs (*n* = 968, 654, 1,152). Gray lines show mean for WT1 to WT4. (*F*) Frequency distribution of τ_O2_ for HS and HE blood. (*G*) Mean ± variance for τ_O2_ and (*H*) overall fluorescence ratio response (*n* = 834 to 1,811) for samples obtained from anemic and compensated HS patients and the compensated HE patient. Gray lines show mean for WT1 to WT4. (*I*) Blood from patient HS1. Cells were loaded with Deep-Red and Green (to measure O_2_ saturation) and SYTO45 (to stain nucleic acids). Overlay of Deep-Red and SYTO45 fluorescence (excited at 633 and 458 nm, respectively). Cells with high SYTO45 fluorescence and O_2_-sensitive Deep-Red/Green ratio are identified as reticulocytes (arrows). Measurements of τ_O2_ and cell area (in the horizontal plane) related to SYTO45 fluorescence. Mean ± SEM (*n* = 1,656, 52, 23, 12). Reticulocytes (Rets) manifest the fastest O_2_ unloading rates. * denotes significant difference to cells with lowest SYTO45 signal (*P* < 0.05). (*J*) Distribution of cell area measured in the horizontal plane for HS RBCs from all six patients studied gated by τ_O2_. (*K*) Relationship between osmolarity and τ_O2_ for HS blood. Mean ± variance (*n* = 1,811, 1,267, 980). (*L*) Relationship between cell half-height and τ_O2_ for WT and HS matched for comparable MCHC. (*M*) Relationship between cell half-height and τ_O2_ for HbH and WT RBCs that have been hypoosmotically swollen to match the hypochromia in HbH (NaCl reduced by 25 mM).

Data obtained from wild-type RBCs subjected to osmotic manipulations of cell thickness (diffusion pathlength) and MCHC (diffusive tortuosity) demonstrate that the cytoplasm imposes a barrier to gas transport. In these experiments, changes to MCHC and cell dimensions are constrained mechanistically (Fig. 5*A*). To investigate other combinations of these variables, blood samples were obtained from patients with hematological disorders. In HbH thalassemia, low MCV and low MCHC are both predicted to favor faster O_2_ unloading. In contrast, RBCs from HS or hereditary elliptocytosis (HE) patients tend to have expanded diffusion paths. O_2_ exchange was measured in blood samples from three HbH patients (HbH1, -2, -3), six HS patients (HS1, -4, -5, -6, -8, -9), and one HE patient. The HS cohort recruited to this study included mutations that produce defects in the membrane skeleton (*SPTB*; 15 to 30% of all HS cases) and those leading to a membrane-destabilizing effect (*SLC4A1*; 33% of all HS cases) (21, 23) (*SI Appendix*, Table S1). Exemplar traces from one HbH sample and one HS sample are shown in Fig. 5*B*.

In HbH thalassemia, the time constant for O_2_ unloading was faster than in wild-type RBCs (Fig. 5 *C* and *D*), consistent with a looser macromolecular environment for O_2_ diffusion and shorter diffusion distance. The surface membrane of HbH RBCs is characterized by greater rigidity (19), which generally reduces the membrane permeability to small solutes (20). The 30% faster τ_O2_ thus indicates that membrane permeation is unlikely to be rate limiting, supporting the notion that cytoplasmic diffusivity is a major determinant of gas exchange. Consistent with the reduction in MCHC, the overall fluorescence ratio change in HbH thalassemia was smaller than in wild-type cells (Fig. 5*E*).

O_2_ exchange in HS and HE RBCs was significantly slower than in wild-type cells (Fig. 5 *F* and *G*). Strikingly, the frequency distribution of τ_O2_ in HS/HE RBCs was wider and asymmetrical (Fig. 5*F*), with a significant fraction of cells manifesting radically slow exchange. Slower τ_O2_ in HS RBCs is unlikely to relate to a change in membrane transport capacity because this was comparable with wild-type cells when probed in terms of AE1 activity (*SI Appendix*, Fig. S5). Instead, the most convincing explanation for slower exchange is the dilated diffusion path due to spherical remodeling (+25% in HS) (*SI Appendix*, Table S1). Moreover, this is numerically consistent with the ∼50% slowing of mean τ_O2_ (diffusion delay is proportional to the square of distance). It is noteworthy that samples from clinically anemic HS patients (mean blood [Hb] = 89 g/L) produced a narrower distribution of τ_O2_ and a faster average compared with compensated patients (mean blood [Hb] = 142 g/L; *P* = 0.0006). In contrast to these marked differences in O_2_ unloading kinetics, the net change in fluorescence ratio was comparable across the cohort of HS patients (Fig. 5*H*), and compensated patients had only modestly higher MCHC (353 vs. 339 g/L; *P* = 0.1) (*SI Appendix*, Table S1). The difference between compensated and anemic HS blood could not be explained by the percentage of reticulocytes (7.8 vs. 10.3%; *P* = 0.49). Instead, the wider distribution of τ_O2_ and shift toward a slower mean in the compensated state are likely related to a greater retention of slowly exchanging spherocytes. These cells may have been more prone to removal in anemic HS patients, explaining the reduction in hematocrit and milder impairment of O_2_ unloading.

Spherical remodeling in HS is a progressive process that first becomes evident in reticulocytes (37). Older RBCs will have a greater degree of spherical remodeling, predicting age-related changes in O_2_ unloading. The youngest circulating RBCs (i.e., reticulocytes) were identified by staining for nucleic acids using SYTO45, which emits fluorescence (480 nm) in a range that is spectrally resolvable from CellTracker Deep-Red, and its excitation wavelength (458 nm) does not excite CellTracker Green. Reticulocytes can be identified by a positive SYTO45 signal and O_2_-sensitive Deep-Red/Green ratio (the latter criterion excludes Hb-negative white blood cells) (*SI Appendix*, Fig. S6*A*). The extent of spherical remodeling can be estimated from cell area measured in the horizontal plane, which decreases as a cell bulges toward a sphere. Data for τ_O2_ and cell area were plotted as a function of SYTO45 fluorescence (patient HS1) (Fig. 5*I*). Compared with mature RBCs, reticulocytes have the largest cell area, demonstrating that they had undergone the least spherical remodeling in circulation. In agreement with the diffusion model, τ_O2_ was fastest in reticulocytes (Fig. 5*I* and *SI Appendix*, Fig. S6*C*). This relationship between diffusion pathlength and τ_O2_ was also shown in second sample (HS6) (*SI Appendix*, Fig. S6*D*). To confirm that gas exchange is slower in more spherically remodeled cells, data pooled from all six HS samples were grouped according to τ_O2_ and reanalyzed in terms of cell area. For this analysis, the τ_O2_ cutoff was 1.6 s because a third of HS cells exchange more slowly than this threshold compared with only a small minority (∼2%) of wild-type RBCs. In agreement with the proposed model, cells with slower τ_O2_ had undergone more substantive spherical remodeling (Fig. 5*J*).

Another consequence of spherical remodeling is that osmotic disturbances will affect cell size in all dimensions, whereas wild-type RBCs tend to increase primarily in thickness (Fig. 3*A*). Consequently, changes in diffusion pathlength are more pronounced in disk-shaped cells, which is why τ_O2_ is slower under hypoosmotic condition, despite the concurrent dilution of MCHC (Fig. 3*C*). In the case of RBCs from compensated HS patients, mild hypoosmotic swelling hastened τ_O2_ (Fig. 5*K*) without greatly affecting the width of the frequency histogram (*SI Appendix*, Fig. S7*A*). This acceleration of τ_O2_ is explained by MCHC dilution outweighing the effect of a more muted change in pathlength. As expected, hyperosmotic shrinkage of HS cells further slowed gas exchange due to a greater tortuosity imposed by Hb crowding. The distinct responses of HS and wild-type RBCs to osmotic changes can be predicted mathematically (*SI Appendix*, Fig. S7*B*).

In summary, disorders involving a change in pathlength (cell size) and tortuosity (MCHC) can substantially influence gas exchange. The positive relationship between τ_O2_ and diffusion pathlength is demonstrated by comparing cells of matching MCHC, such as wild-type and HS RBCs (Fig. 5*L*). A similar relationship can be mapped for HbH cells and osmotically swollen wild-type RBCs at empirically matched MCHC (25 mM reduction in [NaCl]) (Fig. 5*M*).

### Physiological Consequences of Slow Diffusion on Gas Exchange Function.

Measurements of O_2_ unloading from isolated RBCs suggest that gas exchange in capillaries may not attain completion under certain circumstances, such as shortened transit with high perfusion rates. Furthermore, any disease-related amplification of diffusive barriers could render gas exchange inefficient even under normal perfusion. To explore the physiological significance of restricted gas diffusion, the measured index of kinetics (τ_O2_) was converted to the time required to unload 95% of stored O_2_ (T_95_), a more intuitive reporting standard, which is related mathematically as T_95_ = −log(0.05) × τ_O2_. To reflect the physicochemical setting of RBCs in situ, this parameter was corrected for temperature and the presence of CO_2_, which affects Hb–O_2_ binding affinity. To adjust for temperature, T_95_ is scaled by 0.774 as determined experimentally (Fig. 2*B*). To account for typical blood P_CO2_ (40 mmHg), Hb–O_2_ binding kinetics were corrected for modestly reduced O_2_ affinity, modeled to be a factor of 0.949 (30). A requirement of the O_2_ unloading protocol is that the imaged RBCs remain attached to a coverslip, restricting gas exchange to the solution-facing side of cells. Thus, an additional correction was implemented to account for exchange taking place across the entire surface in vivo. Making both sides of the RBC available for exchange has the effect of reducing pathlength by a factor of two (i.e., a fourfold reduction in time delay according to Fick’s law).

**Fig. 6.**
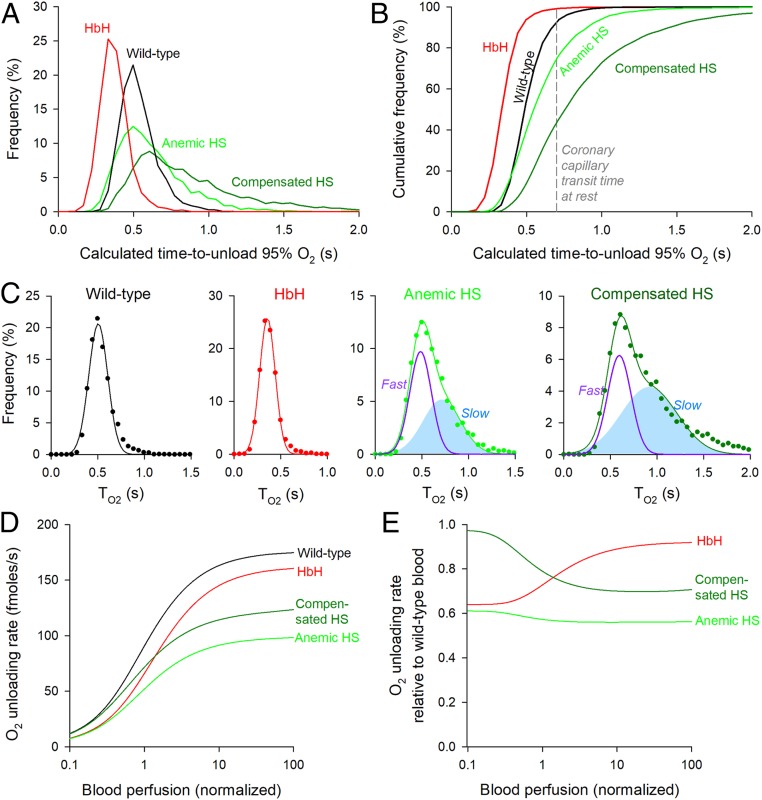
Physiological consequences of slow gas diffusion in RBC cytoplasm. (*A*) Frequency histogram of the time to unload 95% O_2_ (T_95_) from wild-type, HS, and HbH RBCs after correcting for temperature and CO_2_. (*B*) Cumulative frequency distribution. The dashed line denotes typical coronary capillary transit time. (*C*) Analysis of the frequency distribution with mixed Gaussian modeling for wild-type, HbH, anemic, and compensated HS RBCs. Note that, for HS blood, the best fit required two Gaussian curves (fast and slow subpopulations). (*D*) Mathematical simulation of the rate of O_2_ unloading from wild-type, HbH, and HS blood over a range of perfusion rates. (*E*) O_2_ unloading from HbH and HS blood relative to wild-type blood.

Fig. 6*A* compares the distribution of T_95_ for RBCs obtained from wild-type, HbH, anemic HS, and compensated HS patients. The corresponding cumulative frequency curves (Fig. 6*B*) show the proportion of cells able to unload >95% of O_2_ in a given window of time. During the typical transit time of 0.71 s under resting perfusion (38) in a 500-µm-long coronary capillary (39), essentially all HbH RBCs and a 93% majority of wild-type RBCs are expected to unload 95% of stored O_2_. The equivalent percentage among HS RBCs is ∼75% for anemic blood and as little as ∼44% for compensated blood, indicating significantly impaired gas exchange function. The statistical distribution of T_95_ among wild-type and HbH RBCs is adequately described by a single Gaussian (wild type: mean 0.51 s, variance 0.10 s; HbH: mean 0.36 s, variance 0.08 s). In contrast, T_95_ in HS RBCs is best fitted by the sum of two Gaussian curves, representing a faster population and a slower population (Fig. 6*C*). The fraction of cells in the slower subpopulation increased from 48% in anemic HS blood to 62% in compensated HS blood.

The physiological fitness of these RBC populations can be compared using a mathematical model of capillary flow that simulates the rate of O_2_ release as a function of blood perfusion (equations in *SI Appendix*). O_2_ delivery from wild-type blood reaches a plateau of ∼160 fmol/s when perfusion is an order of magnitude above resting. Both HbH and HS blood delivered O_2_ at an impaired rate (Fig. 6*D*). In the case of HbH thalassemia, O_2_ delivery was ∼35% reduced at low perfusion because of reduced blood [Hb], but as perfusion accelerates, the disadvantage relative to wild-type blood became less apparent (Fig. 6*E*). This partial recovery arises because HbH cells are faster at unloading O_2_ and during shorter transit times, can release a greater fraction of stored O_2_ compared with wild-type RBCs. This kinetic advantage partly offsets the effect of reduced MCHC. O_2_ delivery from anemic HS blood was also compromised (by ∼38%) due to low [Hb] and remained significantly disadvantaged at higher perfusion (Fig. 6 *D* and *E*). In compensated HS blood, the nominal recovery of hematocrit supported near-normal O_2_ delivery at low perfusion rates, but this progressively worsened at higher blood flows (Fig. 6 *D* and *E*). This perfusion-dependent impairment to O_2_ delivery can be attributed to the failure of the slower RBC subpopulation to release sufficient O_2_ during abbreviated capillary transit. The above modeling results illustrate that a full recovery of hematocrit does not necessarily predict a full recovery of O_2_ exchange function.

## Discussion

Using single-cell approaches, we have shown that gas handling by RBCs is slower than previously described, which we attribute to a hitherto underestimated effect of restricted diffusion across RBC cytoplasm. To attain the necessary resolving power for tracking O_2_ exchange in an individual RBC, we developed a method that combines microfluidics (to manipulate gas tension) and ratiometric fluorescence imaging (to monitor the ensuing cellular response) (Fig. 1). A consequence of slow gas diffusion is impaired gas unloading at tissues, particularly during periods of increased perfusion, such as exercise (Fig. 6*D*). This finding has clinical implications because disorders of RBCs affecting shape, size, or Hb concentration will strongly influence overall gas exchange.

Our observations were made on RBCs superfused under modest shear rates (γ) of ∼10^2^ s^−1^, which are representative of large vessels (e.g., aorta) (40, 41). In vivo, RBCs traveling through capillaries will deform to fit through the narrow lumen (42), at which point γ can reach 1,500 s^−1^ (27). With rising shear, RBCs first become cup-shaped stomatocytes (or parachutes) and above γ > 400 s^−1^, attain a polylobed shape (40). However, even at γ = 2,500 s^−1^, RBCs do not stretch substantially (43), meaning that their critical thickness remains a significant diffusional barrier, not dissimilar to that in discocytes. Early experimental designs based on highly viscous media (to increase γ) reported tank treading, which results in elliptically stretched (i.e., thinner) RBCs (44, 45). However, tank treading is not normally observed in media of physiological viscosity (27, 41, 46) or in narrow vessels at high γ (41). Although our method does not recapitulate the shear rates in capillaries, it stabilizes cell thickness at a level that is relevant to physiologically deformed cells for robust measurements of diffusional delays.

Our measurements of O_2_ unloading indicate that membrane permeation and cytoplasmic diffusion collectively reduce O_2_ diffusivity in wild-type RBCs to <70 µm^2^/s (i.e., a >35-fold decrease relative to plasma) (3, 29). Based on several independent lines of evidence, we show that the majority of this restriction is attributable to slow cytoplasmic diffusion rather than slow membrane permeation. We first show that CO_2_ diffuses at ∼50 µm^2^/s in the cytoplasmic compartment (Fig. 4*D*). To arrive at this value, we applied a previously published method (14) that probes the dissipation of cytoplasmic solute gradients at the subcellular level and therefore, is not sensitive to membrane properties. Given that O_2_ and CO_2_ have similar diffusive properties in plasma, a substantial resistance to gas transport must reside in the cytoplasm. Using osmotically diluted wild-type cells and RBCs from individuals of low MCHC, we show that this cytoplasmic barrier arises from macromolecular crowding because CO_2_ diffusivity halves with every 62-g/L rise in MCHC (Fig. 4*G*).

A separate line of evidence in favor of rate-setting cytoplasmic diffusion is the response of O_2_ unloading to changes in diffusion pathlength. Osmotic swelling of wild-type RBCs decreases the rate of O_2_ unloading (Fig. 3*C*), despite a concurrent dilution of Hb and a general increase in membrane permeability properties. The latter was probed by analyzing acetic acid entry across the lipid bilayer (Fig. 3*I*), aquaporin-mediated glycerol transport (Fig. 3*F*), and AE1-dependent Cl^−^/OH^−^ exchange (Fig. 3*L*). Although the permeability of these solutes will be different to O_2_, these measurements provide readouts of how the distinct routes for gas entry (the lipid bilayer and gas channels) may respond to osmotic stretch. In all three cases, stretch increased conductance, which is also a likely response of O_2_ permeation. The overall slowing of gas exchange in hypotonically swollen RBCs must, therefore, relate to the expanded diffusion path in swollen cytoplasm. The importance of pathlength was also demonstrated in RBCs from HbH thalassemia and HS patients (Fig. 5). Compared with wild-type RBCs, O_2_ unloading from HbH cells was faster (Fig. 5 *C* and *D*), which our model can explain in terms of a shorter diffusion path acting in concert with lower MCHC (less tortuous cytoplasm). The argument for rate-setting cytoplasmic diffusion is strengthened by the observation that HbH cells also have higher Hb–O_2_ affinity (18) and a more rigid (19) [hence, less permeable (20)] surface membrane, two factors that would have reduced gas exchange kinetics, if rate limiting. HS blood contains RBCs manifesting a range of geometrical remodeling, and the most spherical cells (i.e., bearing the longest cytoplasmic pathlength) were found to exchange gases slower (Fig. 5*J*). In a sample of HS blood, O_2_ unloading was fastest in reticulocytes (i.e., the youngest RBCs in circulation) (Fig. 5*I* and *SI Appendix*, Fig. S6). Compared with mature RBCs, reticulocytes have modestly greater volume (+20%) (37), but the majority of this difference is transmitted to a growth in horizontal cell area (+40%) (Fig. 5*I*). Consequently, HS reticulocytes are thinner than mature RBCs, consistent with the notion that they had the least time in circulation to undergo spherical remodeling. Additionally, reticulocytes have modestly lower MCHC (−15%) (*SI Appendix*, Fig. S6*B*) (37), which also contributes toward faster gas exchange, in agreement with our proposed model. The prominence of the cytoplasmic barrier in setting gas exchange is illustrated by the Fickian relationship between diffusion distance and time delay plotted for cells at matching MCHC (Fig. 5 *L* and *M*).

Overall, our evidence is not supportive of rate-limiting membrane permeation. For instance, if the membrane was rate limiting for O_2_ unloading, then we would have expected faster rates in osmotically swollen wild-type RBCs (due to an activation of membrane permeability properties) (Fig. 3) and slower exchange in HbH cells [due to increased rigidity (19, 20)], but neither of these were observed. While we confirm that blockers of putative gas channels (Hg^2+^/DIDS) slow O_2_ unloading, we attribute this effect to an expansion of pathlength and further slowing of cytoplasmic diffusivity (*SI Appendix*, Fig. S4) rather than a change in membrane permeability. The sensitivity of gas transport to cell thickness highlights the importance of accounting for cell dimensions when interpreting measurements of gas handling in response to drugs or in disease models. Moreover, a cell membrane can be erroneously attributed a low permeability constant if it is adjacent to a high-resistance cytoplasmic barrier, but the measurement technique assumes fast diffusion in cytoplasm.

Given that our method for tracking O_2_ exchange has single-cell resolution, we have been able to describe populations of cells in terms of a tangible measure of RBC physiological fitness. Frequency histograms can readily identify subpopulations, which would otherwise be disguised in pooled population measurements. While wild-type and HbH RBCs are a relatively homogenous population, results from HS patients present evidence for a large subpopulation of spherocytes with significantly impaired gas exchange (Fig. 6*C*). This slow population was more evident in HS patients with compensated [Hb], which could not be explained in terms of the percentage of reticulocytes (*SI Appendix*, Table S1) or a change in the transport capacity of the membrane (probed in terms of AE1 activity) (*SI Appendix*, Fig. S5). Instead, the likely reason for the broader distribution of τ_O2_ and shift toward a lower mean is that the compensated state arises from a retention of spherocytes with longer diffusion pathlengths. Thus, the recovery of hematocrit comes at the price of a slower population-averaged rate of O_2_ unloading. Since these spherical RBCs also have altered deformability (47), gas exchange may be further compromised at the high shear rates inside capillaries.

Findings described herein can influence clinical practice by providing information about the functional quality of blood, an assessment that is lacking in clinical trials and guidelines regarding treatments and procedures, such as splenectomy. Changes to gas transport in disorders of blood are largely interpreted in terms of hematocrit, and consequently, clinical guidelines for anemias generally prioritize interventions that increase RBC count (48), even if this inadvertently leads to a retention of physiologically inferior cells. However, a full recovery of hematocrit may not necessarily produce a proportional improvement in O_2_ delivery as this critically depends on the diffusion delays in cells that underpin the reversal of anemia. The rationale for splenectomy, for instance, is to improve anemia by systemically withholding RBCs from premature destruction. While splenectomy is generally advocated in transfusion-dependent HS [European Hematology Association (49)], the recommendations for moderate forms of the disease (two-thirds of patients) are less clear because of the lack of appropriate randomized trials (49, 50). While there is evidence that splenectomy can ameliorate anemia assayed in terms of O_2_ storage (total [Hb] or hematocrit) (51–53), its effect on gas exchange kinetics is uncharted. Without such a functional assessment, it is not possible to determine whether a splenectomy is physiologically curative. This information would be important in cost–benefit analyses, where infection and thrombosis are major complications of splenectomy (48, 49). As an illustration of the inadequacy of current guidelines, half of children who underwent partial splenectomy were later found to require a full procedure to resolve symptoms (49). This observation may suggest that contemporary recommendations may overestimate the curative value of a partial splenectomy. Future guidelines could improve predictive power if based on prospective studies that factor the gain in the physiological quality of blood against the risks. Analysis of our nonsplenectomized patient cohort reveals a sizable proportion of RBCs unable to provide efficient gas delivery (Fig. 6*B*). Even in HS patients with nominally compensated [Hb] levels, O_2_ exchange kinetics remain below normal. A full restoration of O_2_ delivery rate would, therefore, require an overshoot of hematocrit, which may have unfavorable repercussions (e.g., increased blood viscosity) and possibly necessitate a more radical splenectomy. By monitoring the extent of anemia and obtaining the population-wide distribution of RBC quality, it would be possible to predict the extent to which a partial or complete splenectomy improves physiological outcomes. Such information may provide insights into the hitherto unexplained variability of HS patient responses to splenectomy (54).

In summary, we describe an approach for interrogating the physiological quality of RBCs. We use this to identify barriers to gas flow, characterize population-level heterogeneity in terms of gas exchange kinetics, and evaluate the functional consequences of hematological disorders with single-cell resolution. By providing a kinetic appraisal of blood quality, this method could be implemented in clinical trials that inform guidelines for the clinical management of hematological disorders. Similarly, it could perform quality control of blood for transfusion that is known to undergo spontaneous shape changes during storage (55). The notion of restricted gas diffusion in a protein-rich cytoplasmic matrix is likely to apply to cells other than RBCs. The genesis of tissue hypoxia, which evokes the virtually ubiquitous hypoxia-inducible factor (HIF)-dependent signaling cascade (56), is typically considered in terms of high metabolic O_2_ consumption, but our results indicate that slow O_2_ diffusion may be an additional biologically regulated variable to consider.

## Materials and Methods

### Patients and Ethical Approval.

Venous blood was obtained from healthy volunteers and from consenting patients with hematological disorders (HbH thalassemia, HS, HE, pyruvate kinase deficiency) attending the hematology clinic at Oxford University Hospitals (ethical approval REC 13WA0371). Samples were deidentified prior to use and then, referred to using an anonymized alphanumerical code. Blood parameters were determined by routine clinical methods (Sysmex XN series) and are listed in *SI Appendix*, Table S1. Cell volume was measured flow cytometrically and calibrated by standard procedures. Cell area was measured in CellTracker-labeled RBCs by fluorescence imaging in the horizontal plane during superfusion experiments. Area was calibrated by fluorescent beads (Beckman FluoroSphere) of known geometry (10 µm in diameter). Mean thickness of cells (also referred to as cell height) was calculated as the ratio of MCV to cell area.

### Single-Cell O_2_ Saturation Imaging.

Blood was first diluted 200-fold in Ca^2+^-free Hepes-buffered Tyrode, and cells were loaded with a mixture of CellTracker Deep-Red (5 µM) and CellTracker Green (15 µM) in dimethyl sulfoxide (DMSO). For some experiments, cells were also loaded with SYTO45 (Invitrogen) at 1:1,000 dilution of 5 mM stock. After allowing 10 min for dye loading, cells were spun down, resuspended in Ca^2+^-containing solution, and plated on a poly-l-lysine pretreated Perspex superfusion chamber that was mounted on a Leica LCS confocal system. Cells were superfused with solutions at 23 °C or 37 °C. CellTracker Deep-Red and Green dyes were excited simultaneously by 488- and 633-nm laser lines, and fluorescence was acquired by two photomultiplier tubes at 500 to 550 and 650 to 700 nm at 7.8 Hz in bidirectional *x*–*y* scan mode (128 × 128 pixels) to maximize temporal resolution with pinhole at 4 Airy units to maximize signal to background ratio. Fluorescence was ratioed and normalized to starting ratio.

### Ultrarapid Solution Switching.

A dual microperfusion device (52) released one of two types of solution (57, 58). One microstream was fed from a reservoir bubbled with O_2_; the second microstream was fed from a reservoir containing 1 mM sodium dithionite and bubbled with N_2_ to produce anoxia. Under gravity feed, solution flows were first adjusted manually to produce equal velocity; this was visualized as a sharp interstream boundary when both solutions were running (57, 58). On average, flow per channel was 8 µL/s. At the outlet of the device (square bore of side 400 µm), the linear velocity was 50 mm/s, producing shear rates of 10^2^ s^−1^. At this speed, solution exchange is rapid but does not produce visible deformation of cells as determined by symmetrical cell area and normal cell thickness. Before measurements, the device was flushed to clear stagnant solution, which may have become oxygenated prior to experiments. Following this, the device was placed upstream within a 200 × 200-µm field of view containing RBCs. After equilibration with the oxygenated microstream, O_2_ unloading was triggered by a 30-s exposure to the anoxic microstream.

### Cytoplasmic pH Imaging and H^+^ Uncaging.

Blood was first diluted 200-fold in Ca^2+^-free Hepes-buffered Tyrode, and cells were loaded with the pH reporter cSNARF1 (acetoxymethyl ester; 20 µM). After allowing 10 min for loading, cells were plated on a poly-l-lysine pretreated Perspex superfusion chamber, which was mounted on a Zeiss LSM 700 confocal system. Cells were superfused with Ca^2+^-containing solutions heated to 37 °C. cSNARF1 was excited at 555 nm, and emission was collected at 580 and 640 nm by *x–y* mode scanning and pinhole at 6.3 Airy units. The fluorescence ratio was converted to pH using a previously determined calibration curve (59). To uncage H^+^ ions, 1 mM NVA was added to superfusates, and 405-nm laser light was guided to photolytically release H^+^ ions in a region of interest (ROI) at one end of an RBC (width equal to 1/10th of cell diameter). To produce a continuous source of localized acid loading, uncaging alternated with cSNARF1 imaging every 0.133 s.

### Solutions.

Hepes-buffered solutions were 130 mM NaCl, 4.5 mM KCl, 1 mM CaCl_2_, 1 mM MgCl_2_, 11 mM glucose, and 20 mM Hepes pH adjusted to 6.8, 7.4, or 7.8 with 4 M NaOH. CO_2_/HCO_3_^−^-buffered solutions at pH 7.8 were 75 mM NaCl, 4.5 mM KCl, 1 mM CaCl_2_, 1 mM MgCl_2_, 11 mM glucose, and 55 mM NaHCO_3_ bubbled with 5% CO_2_/balanced air. To change osmolarity, the amount of NaCl was varied, and final osmolarity was measured by freezing point depression (Camlab Osmometer). For osmolarities of 180, 209, 238, 267, 297, 326, 355, and 414 mOsm/kg, NaCl values in Hepes-buffered solutions were 65, 81.25, 97.5, 113.75, 130, 146.25, 162.5, and 195 mM. The concentration of NaCl was reduced accordingly in CO_2_/HCO_3_^−^-buffered solutions (by 55 mM) or in acetate-containing solutions (by 65 mM). For Ca^2+^-free solution, 1 mM Ca^2+^ was replaced with 0.5 mM ethylene glycol bis(b-aminoethyl ether)-*N*,*N*,*N′*,*N′*-tetraacetic acid. All salts were obtained from Sigma-Aldrich.

### Data Availability.

All relevant data are included herein or in *SI Appendix*. All relevant clinical information concerning patient samples is given in *SI Appendix*, Table S1.

## Supplementary Material

Supplementary File
