# Supplementary material for: Single-cell O2 exchange imaging shows that cytoplasmic diffusion is a dominant barrier to efficient gas transport in red blood cells
Source: Proc Natl Acad Sci U S A. 2020 Apr 22;117(18):10067–78. doi: 10.1073/pnas.1916641117 (PMC7211990; doi:10.1073/pnas.1916641117)
Supplement: Supplementary File [file pnas.1916641117.sapp.pdf]

SUPPLEMENTAL TABLE

Table S1: Analysis of red blood cell parameters. Hb: blood hemoglobin concentration; Hct: hematocrit; MCHC: mean corpuscular hemoglobin concentration, MCV: mean corpuscular volume. Cell height is calculated from the quotient of MCV and cell area. Nd – not determined.

Donor	Sex	Age (yrs)	Condition	Hb (g/l)	Hct (ratio)	MCHC (g/l)	Reticulo-cytes (%)	MCV (fl)	Area (μm^2)	½ height (μm)	O_2 unloading measured?
WT1	F	24	Control (volunteer)	145	0.405	358	1.4%	83.3	48.1	0.866	Yes
WT2	F	28	Control (volunteer)	130	0.366	355	1.4%	91.3	49.5	0.923	Yes
WT3	M	39	Control (volunteer)	152	0.433	351	1.4%	86.8	50.4	0.861	Yes
WT4	M	28	Control (volunteer)	154	0.448	344	1.8%	88.5	49.8	0.889	Yes
**MEAN**				**145.25**	**0.413**	**352**	**1.5%**	**87.48**	**49.4**	**0.885**	
S.D.				***10.87***	***0.036***	***6.06***	***0.2%***	***3.35***	***0.99***	***0.028***	
HbH1	F	51	HbH (3 alpha gene deletions)	90	0.312	288	Nd	65.7	54.2	0.606	Yes
HbH2	M	51	HbH 3 alpha gene deletions)	101	0.346	292	Nd	67.1	49.9	0.672	Yes
HbH3	F	25	HbH (3 alpha gene deletions)	88	0.307	287	Nd	68.8	58.6	0.587	Yes
**MEAN**				**93**	**0.322**	**289**		**67.2**	**54.2**	**0.621**	
S.D.				***7.0***	***0.021***	***2.65***		***1.55***	***4.35***	***0.045***	
t-test *P* HbH v WT				<0.001*	0.011*	<0.0001*		<0.001*	0.19	0.0022*	
HS1	M	36	H.S. (SLC4A1 het c.1369_1376)	148	0.408	363	9.0%	99.0	40.6	1.219	Yes
HS2	F	30	H.S. (SPTB het c.4538_4539delTG)	122.5	0.341	360	9.8%	89.7	43.9	1.020	No
HS3	F	32	H.S. (SPTB het c.4538_4539delTG)	130	0.362	359	9.5%	88.7	46.7	0.950	No
HS4	M	39	H.S. (SLC4A1 c.1522G>A)	135	0.385	351	5.4%	89.2	38.5	1.157	Yes
HS5	M	51	H.S. (SLC4A c.489-9G>A)	94	0.270	348	15.2%	94.4	43.4	1.088	Yes
HS6	M	43	H.S. (SPTB c.647+1G>A)	142	0.409	347	9.1%	91.1	43.2	1.053	Yes
HS7	F	63	H.S. (SPTB het c.3765-1G>A)	94	0.274	343	12%	98.2	49.2	1.002	No
HS8	M	71	H.S. (SLC4A1 c.508dupC)	81	0.239	339	11.4%	90.5	44.3	1.021	Yes
HS9	M	75	H.S. (genotype not determined)	91.5	0.276	331	4.5%	96.0	42.6	1.116	Yes
**MEAN**				**115.33**	**0.329**	**348.94**	**9.5%**	**92.97**	**43.6**	**1.070**	
S.D.				***25.22***	***0.065***	***10.46***	***3.3%***	***4.00***	***3.10***	***0.085***	
t-test *P* HS v WT				0.047*	0.038*	0.61	<0.001*	0.036*	0.0004*	0.0001*	
HE1	F	43	H.E. (EBP41 het c.495C>G p.Tyr154*)	144	0.411	350	Nd	96.7	50.9	0.950	Yes
IDA1	M	35	Iron deficient	117	0.372	315	Nd	81.4	39.9	1.021	Yes
IDA2	F	24	Reduced MCHC, anemic	137	0.435	315	Nd	94.0	48.4	0.971	Yes
IDA3	F	23	Reduced MCHC, not clinically anemic	143	0.439	326	Nd	96.1	52.5	0.933	Yes
PKD	M	51	Pyruvate kinase deficiency	80	0.268	299	Nd	119.1	48.9	1.217	No

www.pnas.org/cgi/doi/10.1073/pnas.1916641117

SUPPLEMENTAL FIGURES

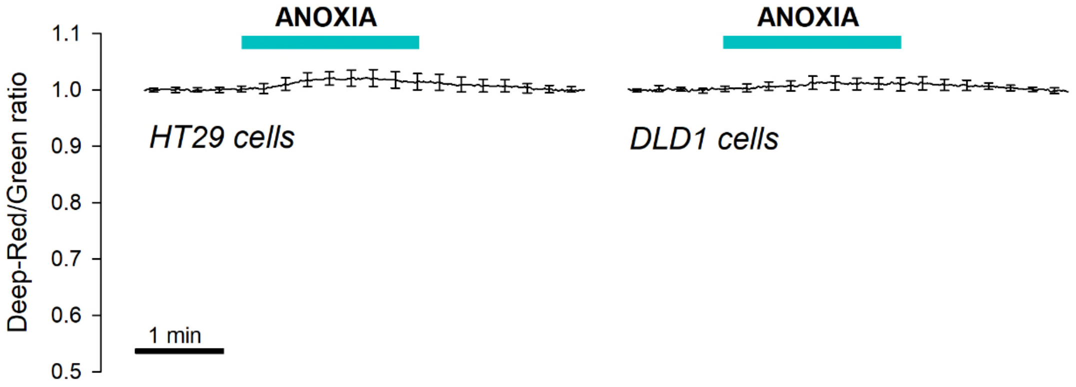

Figure S1: *Fluorescence-ratio response to anoxia is absent in cells lacking hemoglobin.* Two colorectal cancer lines (HT29 and DLD1) were grown on glass coverslips, until confluent, and then re-suspended in culture medium and loaded with Green and Deep-Red, as per the protocol for RBCs. The microstream maneuver, normally performed on RBCs, was applied to cancer cells once these had settled at the bottom of the superfusion chamber. Even a 2 min exposure to the anoxic microstream did not decrease fluorescence ratio, unlike the response in RBCs (typically, a decrease to 0.6 within seconds). Mean±S.D., from 13 DLD1 cells and 37 HT29 cells. These results indicate that chemical hypoxia is not affecting the fluorescent properties of Green or Deep-Red dyes *per se*. Thus, the profound effect on ratio reported in RBCs is attributable to O_2-dependent changes in hemoglobin's absorption of Deep-Red fluorescence.

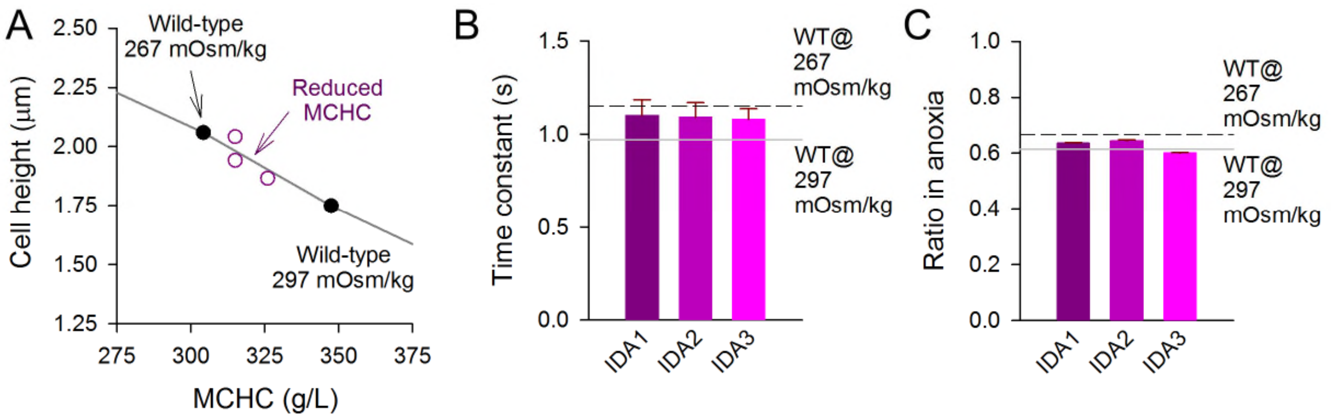

Figure S2: *Measurements on RBCs with reduced MCHC*. **(A)** Blood samples were obtained from three individuals (IDA1-3) with MCHC that was lower than the control cohort, and also had greater RBC thickness. Their MCHC/thickness combinations fall on the relationship empirically derived for wild-type RBCs subjected to a range of osmotic manipulations *(gray line;* replotted from Fig 5A). **(B)** Time constant of O_2 unloading (τ_{O2}) and **(C)** fluorescence ratio change in response to deoxygenation. The average values of these readouts in IDA RBCs fell between wild-type data recorded at normal osmolarity (297 mOsm/kg) and modestly reduced osmolarity (267 mOsm/kg). Slower O_2 unloading in IDA RBCs is consistent with the relationship shown in Fig 3C.

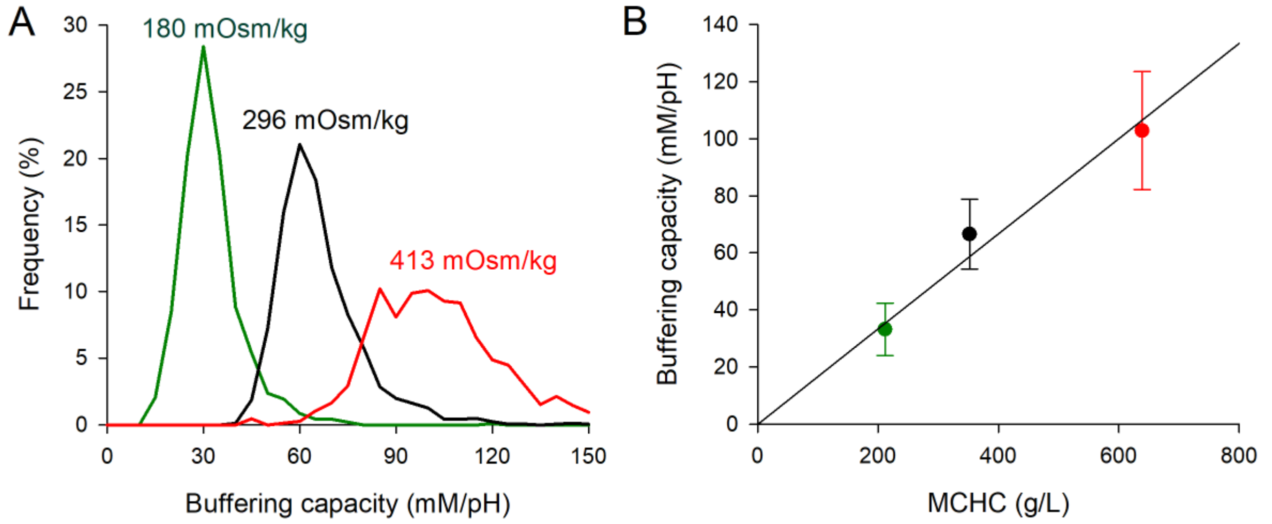

Figure S3: *Buffering capacity in wild-type RBCs.* Intracellular buffering capacity was measured in the absence of CO_2/HCO_3^-. **(A)** Upon exposure of cells to 65 mM Na-acetate, intracellular [acetate] increases and pH_i decreases, and their ratio is a measure of buffering capacity. Since acetic acid partitions uniformly across the membrane, intracellular [acetate] is related to extracellular [acetate] (see Equations). Measurements were repeated for a range of osmolarity. **(B)** Relationship between MCHC (calculated from blood [Hb], haematocrit and MCV) and buffering capacity. Slope is consistent with ~11 moles buffering sites per mole of Hb, in agreement with literature values: Cass & Dalmark (1973) and Dalmark (1975) calculated Hb-dependent buffering to be 53-68 mM/pH at normal osmolarity. The agreement between empirically-measured buffering and buffering calculated for Hb confirms that haemoglobin is the major cytoplasmic buffer in RBCs superfused with CO_2/HCO_3^--free media.

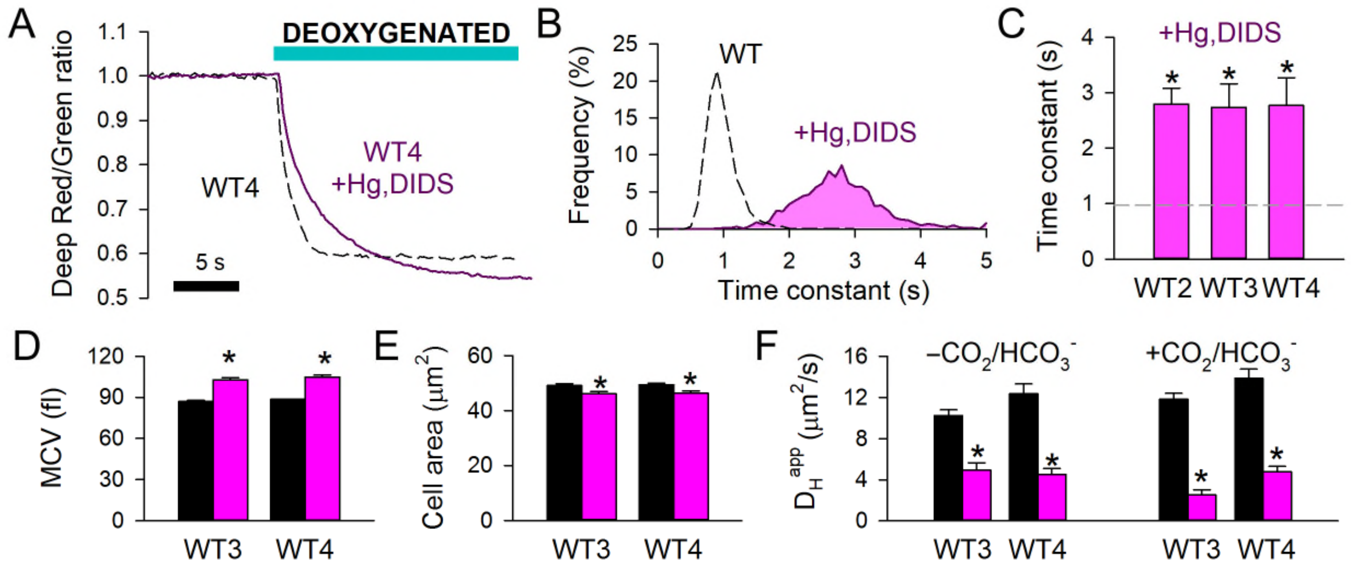

Figure S4: *Effect of mercury on gas transport in wild-type RBCs.* **(A)** O_2 unloading protocol performed on wild-type RBCs pretreated with 250 µM Hg^{2+} and 100 µM DIDS for 10 min prior to imaging. **(B)** Distribution of the O_2 unloading time constant. **(C)** O_2 unloading was 3-fold slower after Hg^{2+}/DIDS treatment. Dashed line shows mean τ_{O2} under control conditions. Mean±variance (N=633, 458, 589). **(D)** MCV measured by flow cytometry (6 repeats). **(E)** Cell area measured in the horizontal plane during imaging (N=102, 130). **(F)** Effect of treatment on D_H^{app} in the presence and absence of CO_2/HCO_3^-. Mean±variance (N=15-25). * significant difference relative to drug-free control (P<0.05).

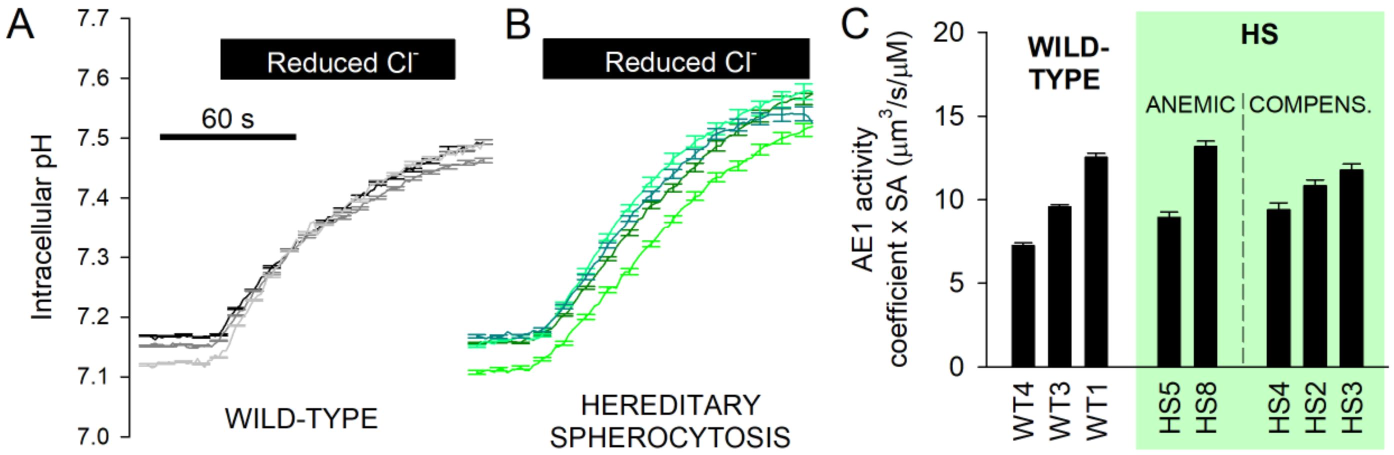

Figure S5: *Measuring AE1 activity in wild-type and HS RBCs.* Net AE1 activity was triggered by reducing extracellular chloride concentration by iso-osmotic substitution with 100 mM gluconate. In the absence of CO_2/HCO_3^- buffer, this solution-manoeuvre triggers Cl^-/OH^- exchange, reported as an intracellular alkalinisation. The initial rate of this response provides an estimate of AE1 activity. **(A)** Experiments performed on wild-type RBCs and **(B)** hereditary spherocytosis RBCs. **(C)** Quantification of the membrane's AE1 transport capacity in terms of the product of activity coefficient and surface area (SA; see Equations). This measure of membrane activity was not significantly different in HS RBCs, relative to wild-type cells (P=0.51). No substantial difference in average AE1 activity was noted between anemic and compensated HS patients.

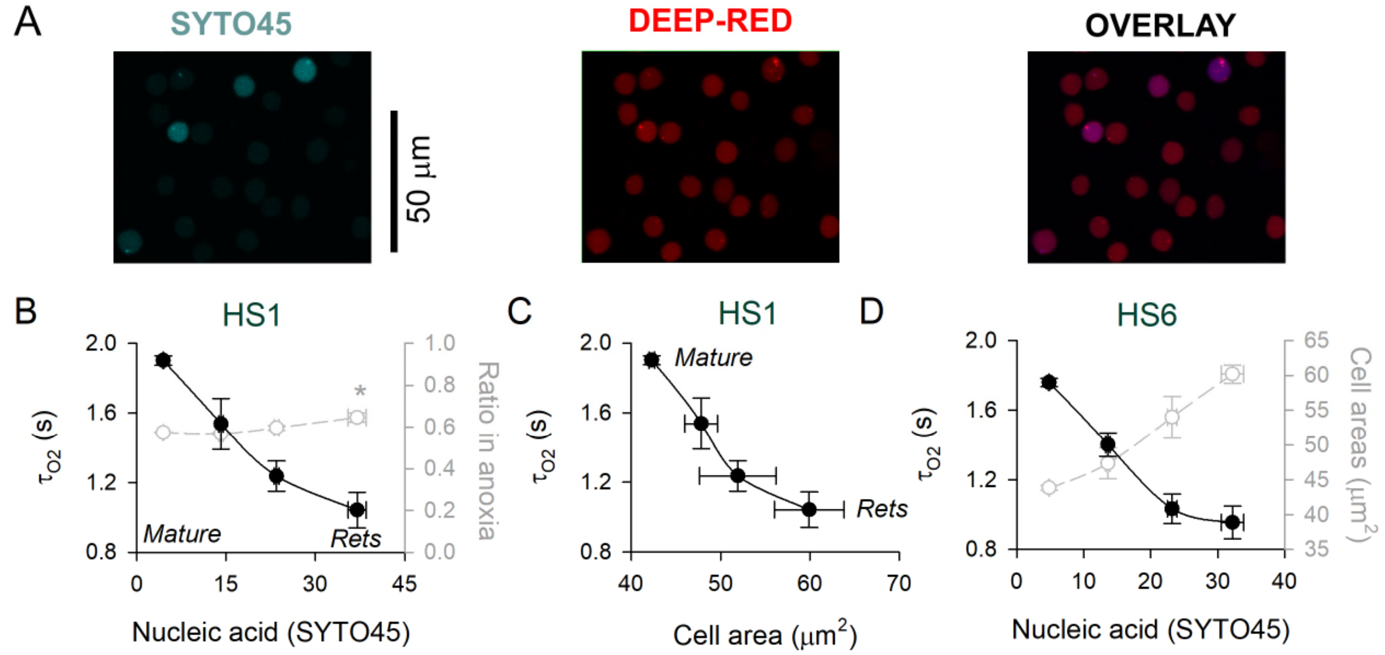

Figure S6: *Measuring O_2 unloading from reticulocytes in HS blood.* **(A)** *Left:* fluorescence image showing SYTO45-positive cells (staining for nucleic acids) in HS1 blood. *Middle:* fluorescence image of CellTracker Deep-Red. *Right:* overlay of Deep-Red and SYTO45; reticulocytes appear purple. **(B)** Blood from patient HS1. Analysis of O_2 unloading time constant (τ_{O2}; left axis) and overall change in DeepRed/Green fluorescence ratio (right axis) as a function of SYTO45 fluorescence. Reticulocytes have higher SYTO45 fluorescence. Only cells containing haemoglobin (i.e. reticulocytes and mature RBCs) respond to anoxia with a change in Deep-Red/Green ratio; non-responding cells (e.g. white blood cells) were excluded from the analysis. **(C)** Data replotted from Fig 5I. Reticulocytes have the largest cell area (measured in the horizontal plane) and fastest (τ_{O2}), as expected from cells that have had the least spherical remodelling in circulation. **(D)** Analysis performed on blood from patient HS6, confirming faster O_2 unloading in reticulocytes.

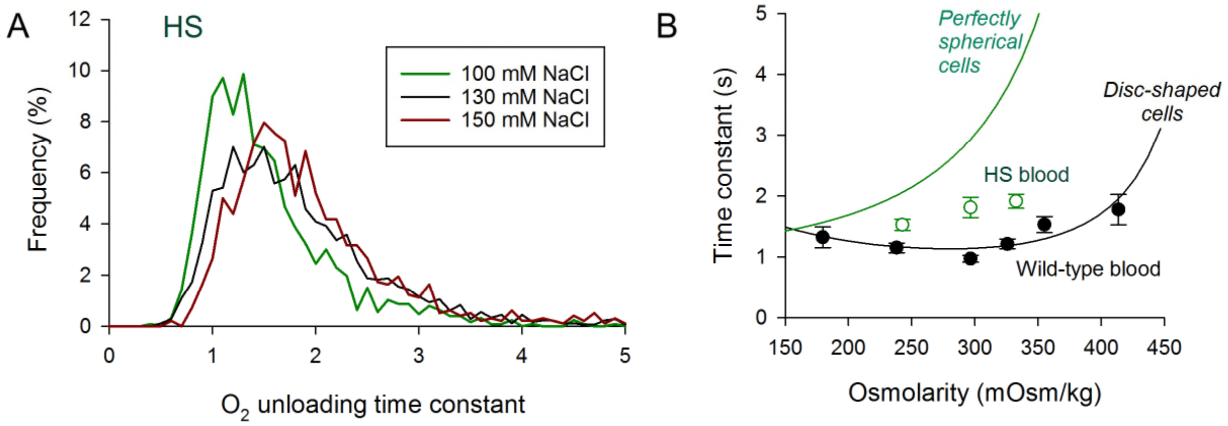

Figure S7: *Response of HS RBCs to changes in osmolarity.* **(A)** Frequency distribution of O_2 unloading time constant (τ_{O2}) for HS blood recorded at normal osmolarity (130 mM NaCl), reduced (100 mM NaCl) and raised (150 mM NaCl) osmolarity. **(B)** Relationship between osmolarity and τ_{O2} for wild-type and HS blood. Superimposed in black is a mathematical fit for wild-type cells, assuming that the rate of unloading is the product of cell half-thickness squared and diffusivity empirically related to MCHC. Applying the same mathematical equations but to a perfectly spherical cell, the model prediction is monophasic. HS cells, which undergo a degree of spherical remodeling, fall between these two extremes.

SUPPLEMENTAL EQUATIONS

(1) Calculating effective O_2 diffusivity from Hb-O_2 kinetics and O_2 unloading time constants (Figure 2I):

The diffusion-reaction process governing O_2 unloading is illustrated schematically in Figure 2H. The equations describing intracellular O_2 concentration (u_1), deoxygenated Hb (u_2) and oxy-hemoglobin (u_3) are:

$$\frac{\partial u_1}{\partial t} = D_{O2}^{eff} \times \frac{\partial^2 u_1}{dz^2} + k_d \times (u_3 - K_{HbO2}(u_1) \times u_1 \times u_2)$$

$$\frac{\partial u_2}{\partial t} = D_{Hb} \times \frac{\partial^2 u_2}{dz^2} + k_d \times (u_3 - K_{HbO2}(u_1) \times u_1 \times u_2)$$

$$\frac{\partial u_3}{\partial t} = D_{Hb} \times \frac{\partial^2 u_3}{\partial z^2} - k_d \times (K_{HbO2}(u_1) \times u_1 \times u_2)$$

Here, D^{eff}_{O2} is the effective diffusivity of O_2 which lumps membrane permeability and cytoplasmic diffusivity into one term, defined by Eq 1. The system of equations was solved using MATLAB's pdepe solver over a one-dimensional space, equivalent to the z axis, i.e. along the height of a RBC. Since RBCs settle at the base of a coverslip, the spatial range was from z=0 (coverslip) to $z=h=1.770$ µm (upper side of RBC exposed directly to superfusate; **Table S1**). The initial condition was defined as a fully oxygenated RBC. All constants from the literature were selected for 23°C. O_2 solubility was 1.7856×10^{-6} M/Torr, thus 100% O_2-saturated solution contained 1.4 mM of O_2. D_{Hb} was obtained from D_H^{app} data obtained in the absence of CO_2/HCO_3^- buffer (**Figure 4E**), 10.4 µm²/s. The boundary condition at $z=0$ µm was set to zero-flux (reflection). At $z=h$, the boundary condition was defined by extracellular O_2, controlled by ultra-rapid switching. Rate constant k_d represents O_2 unbinding from oxy-hemoglobin. Previous reports have measured the rate of unbinding of the first O_2 from oxyhemoglobin to be in the range 20-30 s^{-1} at room temperature (1, 2), but subsequent unbinding steps are faster, due to cooperativity, resulting in an overall unbinding constant approaching ~100 s^{-1}. Thus, k_d was taken as a variable over a restricted physiological range. The second variable in the system was the effective O_2 diffusion coefficient in RBC cytoplasm, D^{eff}_{O2}, which was varied from zero (immobile) to 2500 µm²/s (O_2 diffusivity in water). Hb affinity for O_2 was described by K_{HbO2} which itself was defined as a function of O_2 to account for cooperativity (3). The diffusion-reaction system was run to find pairs of values for k_d and D^{eff}_{O2} that predict an ensemble O_2 unloading time constant (τ_{O2}) of 0.9713 s, i.e. experimentally measured in wild-type RBCs. Thus, it is possible to plot the unique relationship between k_d and D^{eff}_{O2} that are in agreement with experimental measurements of τ_{O2}. Since a range of k_d is restricted, it is possible to derive a range of D^{eff}_{O2}, as shown by the shaded area in Figure 2I.

(2) Calculating membrane permeability to acetic acid and pH buffering capacity (Figure 3):

Upon exposure to 65 mM Na-acetate, intracellular pH (pH_i) acidifies, as intracellular [acetate] builds-up. In the absence of AE1 activity, the system reaches a steady-state. The rise in intracellular [acetate] divided by the change in pH_i (ΔpH_i) is a measure of buffering capacity. At equilibrium, intracellular [acetate] is related to [acetic acid], which partitions equally across the membrane, the acetic acid dissociation constant K_{ac}, and pHi:

$$[acetate]_i = [acetic\ acid] \times \frac{K_{ac}}{10^{-pHi}}$$

The time course of pH_i change, evoked by acetic acid entry, provides a measure of membrane permeability to acetic acid (P_{ac}). Fitting this time course to a monoexponential provides estimates of constants A, B and C:

$$pH_i = A + B \times \exp\left(-\frac{t}{C}\right)$$

The first derivative of this equation provides the rate of change:

$$\frac{dpH_i}{dt} = -\frac{B}{C} \times \exp\left(-\frac{t}{C}\right)$$

Thus, at the start (t=0), the initial rate of change is equal to *–B/C*. When multiplied by buffering capacity, β, this gives flux *J* of acid (acetic acid) across the membrane:

$$J = -\frac{B}{C} \times \beta$$

Flux can be expressed in terms of permeability, the transmembrane [acetic acid] gradient, surface area (SA) and volume (MCV):

$$J = \frac{P_{ac} \times [acetic\ acid]_e \times SA}{MCV}$$

The product of P_{ac} and SA provides an appraisal of the membrane's conductance to acetic acid:

$$\frac{P_{ac} \times [acetic\ acid]_e \times SA}{MCV} = -\frac{B}{C} \times \beta$$

$$P_{ac} \times SA = -\frac{B \times MCV \times \beta}{C \times [acetic\ acid]_e}$$

Since B is equal to ΔpH, and considering the definition of β, this simplifies to:

$$P_{ac} \times SA = \frac{MCV}{C} \times \frac{K_{ac}}{10^{-pHi}}$$

(3) Calculating AE1 activity (Figure 3):

Flux J carried by AE1 is defined thermodynamically in terms of the surface area (SA), volume (MCV), activity coefficient (k, related to the number of AE1 proteins embedded in the membrane), and the intracellular (i) and extracellular (e) concentrations of Cl^- and H^+ ions:

$$J = \frac{SA}{MCV} \times k \times ([H]_e \times [Cl]_e - [H]_i \times [Cl]_i)$$

This flux is related to the rate of change of intracellular pH (pHi) and buffering capacity, β:

$$J = -\frac{dpH_i}{dt} \times \beta$$

Fitting the measured pH_i time course to a monoexponential provides estimates of constants A, B and C:

$$pH_i = A + B \times \exp\left(-\frac{t}{C}\right)$$

The first derivative of this equation provides the rate of change:

$$\frac{dpH_i}{dt} = -\frac{B}{C} \times \exp\left(-\frac{t}{C}\right)$$

Thus, at the start (t=0), the initial rate of change is equal to *–B/C*. Thus,

$$J = -\frac{B}{C} \times \beta$$

The ensemble AE1 transport capacity of the membrane can be described by the product of k and SA, given by:

$$-\frac{B}{C} \times \beta = \frac{SA}{MCV} \times k \times ([H]_e \times [Cl]_e - [H]_i \times [Cl]_i)$$

$$SA \times k = -\frac{B}{C} \times \frac{\beta}{([H]_e \times [Cl]_e - [H]_i \times [Cl]_i)}$$

(4) Solving H^+ diffusion equations (Figure 4):

Full details of the method have been published previously(4). Briefly, the diffusion equation describing the spatio-temporal concentration, u, of H^+ ions,

$$\frac{\partial u}{\partial t} = D_H^{app} \times \nabla^2 u + J$$

was solved using MATLAB's pdetool over a two-dimensional geometry defined by the outline of an RBC imaged in the xy plane with reflection (zero-flux) boundary conditions. Injection of acid J was simulated as a constant-source flux in a region corresponding to the photolysis site. Initial conditions were set to zero, so to simulate the rise in free $[H^+]$. The results of the simulation were averaged in ten regions of interest that correspond to those used for image processing. The best-fit D_H^{app} was inferred by the least squares method between experimental data and simulations, after normalizing cell-averaged time courses.

(5) Obtaining CO_2 diffusivity from spatio-temporal H^+ dynamics (Figure 4):

The solution to a simplified diffusion equation describes D_H^{app}, which is mathematically related to the diffusion coefficients (weighted by buffering capacity) of all intracellular pH buffers (5). The diffusion-reaction process involving these components can be simulated by a system of three equations describing intracellular $[H^+]$ (u_1), $[HCO_3^-]$ (u_2) and $[CO_2]$ (u_3) over a one-dimensional space between x=0 (site of uncaging) and x=L (where L is the length of the RBCs):

$$\frac{\partial u_1}{\partial t} = D_{Hb} \times \frac{\partial^2 u_1}{\partial x^2} + \frac{CA_i \times (k_f \times u_3 - k_r \times u_1 \times u_2) + J(x)}{\beta_{Hb}/(2.303 \times u_1)}$$

$$\frac{\partial u_2}{\partial t} = D_{HCO3} \times \frac{\partial^2 u_2}{\partial x^2} + CA_i \times (k_f \times u_3 - k_r \times u_1 \times u_2)$$

$$\frac{\partial u_3}{\partial t} = D_{CO2} \times \frac{\partial^2 u_3}{\partial x^2} - CA_i \times (k_f \times u_3 - k_r \times u_1 \times u_2) + \rho \times P_{CO2}^{app} \times (u_3^0 - u_3)$$

The rate of H^+ uncaging, J, was equal to the experimentally-determined acidification rate, and restricted to the area of uncaging. Mean cell thickness (h) is calculated as the ratio of MCV to area measured from the cell's fluorescence outline. D_{Hb} is equal to D_H^{app} measured in the absence of CO_2/HCO_3^-. D_{CO2} is related to D_{HCO3} by a ratio of 1.46 (6) k_f and k_r. the uncatalyzed forward and reverse rate constants of CO_2 hydration at 37°C, are 0.18 s^{-1} and 0.23 $\mu M^{-1}\ s^{-1}$ (6, 7). CA_i activity was set to 10^4, i.e. the near-equilibrium condition. Buffering due to hemoglobin (β_{Hb}) is 0.178×MCHC (8, 9). ρ is the surface area-to-volume ratio and approximated as the inverse of cell half-thickness (h/2). P_{CO2}^{app}, the apparent permeability to CO_2 across the thickness of the cell h, is defined as $D_{CO2}/(h/2)$ i.e. diffusion across the mean path-length over the domain 0 to h. Extracellular CO_2 (u_3^0) is 1.2 mM (i.e. 5% CO_2).

The equations were solved using MATLAB' s pdepe solver for initial conditions defined by starting pH_i and CO_2, and boundary conditions were set to zero-flux. Sensitivity analyses indicated that small variations in CA_i had no meaningful effect on outcomes, thus the principal unknown variable that influences H^+ dynamics is D_{CO2}. By solving the mechanistic and simplified diffusion equations, it is possible to pair a value for D_H^{app} with a best-fitting D_{CO2}, as shown by the curves in Figure 1H. Thus, an experimental measurement of D_H^{app} in the presence of CO_2/HCO_3^- buffer can be converted to D_{CO2}.

(6) Simulation of the rate of O_2 delivery at tissues as a function of perfusion rate (Figure 6D/E):

The statistical description of RBC O_2 unloading rates was obtained by fitting the histogram data to Gaussian curves (**Figure 6C**). In the case of wild-type and HbH blood, the populations were best described by a single Gaussian. In contrast, HS blood was best described by two Gaussians, each with a mean, variance and size. An algorithm written in MATLAB calculated the rate of O_2 release during a capillary transit time for a given rate of perfusion, varying between one tenth of resting flow and a 100-fold hyperemia. The capillary geometry was selected from literature values for coronary capillaries (10): length 500 µm and radius 3 µm, hence a volume V_{cap} of 9425 fL. Under resting perfusion, the transit time through such capillaries is 0.71 s (11). Values for MCV and MCHC are listed in **Table S1**. The average number of RBCs, *N*, in the capillary at any one time was obtained from hematocrit and MCV (N=hct×V_{cap}/MCV). Fractional O_2 unloading during a transit time of *T* is estimated by monoexponential decay (1-exp(-T/τ_{O2})). The histogram distribution function, *H,* was used to obtain a weighted mean of O_2 unloading time. Thus, a fully O_2-saturated RBC, which carries 4×MCHC×MCV/64458 fmoles of O_2 per cell, will release an amount of O_2 during transit time *T* given by (units: fmoles O_2 per second):

$$Unloading\ rate = 4 \times \frac{MCHC \times MCV}{64458} \times \frac{N}{T} \times \frac{\sum_\tau H(\tau_{O2}) \times \left(1 - e^{-T/\tau_{O2}}\right)}{\sum_\tau H(\tau_{O2})}$$

ADDITIONAL REFERENCES

1. S. Chakraborty, V. Balakotaiah, A. Bidani, Diffusing capacity reexamined: relative roles of diffusion and chemical reaction in red cell uptake of O2, CO, CO2, and NO. *J Appl Physiol (1985)* **97**, 2284-2302 (2004).
2. J. S. Olson, E. W. Foley, D. H. Maillett, E. V. Paster, "Measurement of Rate Constants for Reactions of O2, CO, and NO with Hemoglobin" in Hemoglobin Disorders: Molecular Methods and Protocols*,* R. L. Nagel, Ed. (Humana Press Inc, Totowa, N.J., 2003), vol. 82, pp. 65.
3. R. K. Dash, B. Korman, J. B. Bassingthwaighte, Simple accurate mathematical models of blood HbO2 and HbCO2 dissociation curves at varied physiological conditions: evaluation and comparison with other models. *Eur J Appl Physiol* **116**, 97-113 (2016).
4. S. L. Richardson, P. Swietach, Red blood cell thickness is evolutionarily constrained by slow, hemoglobin-restricted diffusion in cytoplasm. *Sci Rep* **6**, 36018 (2016).
5. W. Junge, S. McLaughlin, The role of fixed and mobile buffers in the kinetics of proton movement. *Biochim Biophys Acta* **890**, 1-5 (1987).
6. C. Geers, G. Gros, Carbon dioxide transport and carbonic anhydrase in blood and muscle. *Physiol Rev* **80**, 681-715 (2000).
7. A. Hulikova, N. Aveyard, A. L. Harris, R. D. Vaughan-Jones, P. Swietach, Intracellular carbonic anhydrase activity sensitizes cancer cell pH signaling to dynamic changes in CO2 partial pressure. *J Biol Chem* **289**, 25418-25430 (2014).
8. A. Cass, M. Dalmark, Equilibrium dialysis of ions in nystatin-treated red cells. *Nat New Biol* **244**, 47-49 (1973).
9. M. Dalmark, Chloride and water distribution in human red cells. *J Physiol* **250**, 65-84 (1975).
10. G. S. Kassab, Y. C. Fung, Topology and dimensions of pig coronary capillary network. *American Journal of Physiology* **267**, H319-325 (1994).
11. M. F. Allard, C. T. Kamimura, D. R. English, S. L. Henning, B. R. Wiggs, Regional myocardial capillary erythrocyte transit time in the normal resting heart. *Circ Res* **72**, 187-193 (1993).
